# An La-related protein controls cell cycle arrest by nuclear retrograde transport of tRNAs during diapause formation in *Artemia*

**DOI:** 10.1186/s12915-016-0239-4

**Published:** 2016-03-03

**Authors:** Dian-Fu Chen, Cheng Lin, Hong-Liang Wang, Li Zhang, Li Dai, Sheng-Nan Jia, Rong Zhou, Ran Li, Jin-Shu Yang, Fan Yang, James S. Clegg, Hiromichi Nagasawa, Wei-Jun Yang

**Affiliations:** College of Life Sciences, Zhejiang University, Hangzhou, 310058 People’s Republic of China; Key Laboratory of Conservation Biology for Endangered Wildlife of the Ministry of Education, Zhejiang University, Hangzhou, 310058 People’s Republic of China; Key Laboratory of Protein Chemistry and Developmental Biology of the State Education Ministry of China, College of Life Sciences, Hunan Normal University, Changsha, 410018 People’s Republic of China; Tianjin Key Laboratory of Animal and Plant Resistance, College of Life Sciences, Tianjin Normal University, Tianjin, 300387 People’s Republic of China; Section of Molecular and Cellular Biology and Bodega Marine Laboratory, University of California, Davis, Bodega Bay, California 94923 USA; Department of Biological Chemistry, The University of Tokyo, Yayoi, Bunkyo, Tokyo, 113-8657 Japan

**Keywords:** *Artemia*, Cell cycle arrest, Diapause embryo, La-related protein, Nuclear retrograde of tRNA

## Abstract

**Background:**

In eukaryotes, tRNA trafficking between the nucleus and cytoplasm is a complex process connected with cell cycle regulation. Such trafficking is therefore of fundamental importance in cell biology, and disruption of this process has grave consequences for cell viability and survival. To cope with harsh habitats, *Artemia* has evolved a special reproductive mode to release encysted embryos in which cell division can be maintained in a dormancy state for a long period.

**Results:**

Using *Artemia* as a peculiar model of the cell cycle, an La-related protein from *Artemia*, named Ar-Larp, was found to bind to tRNA and accumulate in the nucleus, leading to cell cycle arrest and controlling the onset of diapause formation in *Artemia*. Furthermore, exogenous gene expression of Ar-Larp could induce cell cycle arrest in cancer cells and suppress tumor growth in a xenograft mouse model, similar to the results obtained in diapause embryos of *Artemia*. Our study of tRNA trafficking indicated that Ar-Larp controls cell cycle arrest by binding to tRNAs and influencing their retrograde movement from the cytoplasm to the nucleus, which is connected to pathways involved in cell cycle checkpoints.

**Conclusions:**

These findings in *Artemia* offer new insights into the mechanism underlying cell cycle arrest regulation, as well as providing a potentially novel approach to study tRNA retrograde movement from the cytoplasm to the nucleus.

**Electronic supplementary material:**

The online version of this article (doi:10.1186/s12915-016-0239-4) contains supplementary material, which is available to authorized users.

## Background

Regulation of the cell cycle involves processes crucial to survival, including the detection and repair of genetic damage and the prevention of uncontrolled cell division. This subject has attracted much attention and has enormous practical implications for combating cancer. The cell cycle has several well-controlled phases. Progression into each phase is tightly regulated by checkpoints and inhibitors [1, 2]. The checkpoint allows the cell to repair DNA damage before entering mitosis, and studies of this process may lead to chemotherapies with increased cytotoxicity. Two families, Cip/Kip and INK4a/ARF, prevent progression of the cell cycle. The Cip/Kip family includes p21, p27, and p57, which halt the cell cycle in G1 phase by binding to and inactivating cyclin-CDK complexes [3]. The INK4a/ARF family includes p16^INK4a^, which binds to CDK4 and arrests the cell cycle in G1 phase, and p14^Arf^ in humans, which prevents p53 degradation [1]. Each of these proteins acts through checkpoint signaling pathways that lead to cell cycle arrest [4]. In response to environmental stresses, such as starvation or treatment with thymidine or aphidicolin, cell cycle progress is halted in G1 phase by the control of multiple molecular pathways, rather than a single pathway [5].

In eukaryotes, tRNA trafficking between the nucleus and the cytoplasm is a complex process that often responds to environmental stress, thereby connecting transcription in the nucleus to translation in the cytoplasm [6]. This trafficking is of fundamental importance to cell biology, and disruption of this process has grave consequences for cell viability and survival. In response to genotoxic stress, regulation of tRNA trafficking constitutes an integral physiological adaptation to DNA damage at the cell cycle checkpoint. For example, in the yeast *Saccharomyces cerevisiae*, un-spliced tRNA rapidly accumulates in the nuclei, and cell cycle progression is delayed at the G1 to S phase transition [7, 8]. In contrast to previous findings that showed that the transport of tRNA is unidirectional from the nucleus to the cytoplasm, recent studies have revealed that cytoplasmic tRNAs move retrogradely toward the nucleus and accumulate in response to nutrient deprivation [9–12]. Retrograde tRNA nuclear import might also serve to proofread tRNAs after splicing in order to separate improperly spliced tRNAs from the translation machinery [13]. The regulation of tRNA trafficking is dependent on the subcellular distribution of the elongation factor Tef1/2 (the yeast ortholog of eEF1α). The function of Tef1/2 is required for efficient tRNA nuclear export [14] and is dependent on Msn5 and Mtr10, as is the tRNA retrograde transport pathway [15].

La proteins are confined to the nucleus and the complete evolutionary in eukaryotic cells. Structural analysis of these proteins resulted in their classification into five families, namely the genuine La homologs and four Larp families: Larp1, 4, 6, and 7 [16, 17]. La proteins contain two RNA-binding motifs (RRMs) and an La motif, and have important functions in RNA metabolism. These proteins interact with an extensive variety of cellular RNAs, and exhibit activities to promote the metabolism of nascent pol III transcripts by binding to their common UUU-3’OH-containing ends and to modulate the translation of certain mRNAs involving an unknown binding mechanism [18].

The *Artemia* model studied here is found in severely hypersaline environments. Under certain conditions, mature females produce and release encysted gastrula embryos (also called cysts) that enter diapause, a state of obligate dormancy. Different environmental cues lead to uninterrupted (direct) embryonic development, resulting in the release of swimming nauplius larvae [19]. A feature of diapause embryos that is central to the present study is the complete absence of cell division and DNA synthesis during embryonic diapause [20, 21]. Diapause can be terminated by certain environmental conditions, leading to activated post-diapause embryos [19, 21]. Remarkably, these activated encysted embryos develop without any DNA synthesis or cell division [22], and eventually hatch as nauplius larvae, at which point DNA synthesis and cell division resume [21, 23]. The *Artemia* model depicts adaptation as a complex response to critical life conditions, integrating and refining past and present experiences at all levels of organization [24].

To elucidate the molecular mechanism underlying cell cycle arrest and its link to the regulation of tRNA nucleocytoplasmic trafficking, *Artemia* diapause was used as a cell cycle arrest model. In this study, an RNA-binding and La-related protein, named Ar-Larp, was found to accumulate in the nucleus in response to cell cycle arrest, which resulted in the formation of *Artemia* diapause by binding to tRNAs. The mechanisms underlying the regulation of cell cycle arrest by Ar-Larp were elucidated in cancer cells using exogenous *Ar-Larp* gene transfection and expression. Cell cycle arrest induced by tRNA retrograde movement from the cytoplasm to the nucleus was then demonstrated in cancer cells. Our results indicated that tRNA trafficking regulates the mitogenesis and proliferation of cells through cell cycle checkpoints, a process that is mediated by multiple signaling pathways including histone H3 acetylated at lysine 56 (H3K56ac), extracellular signal-regulated kinase (ERK), and Akt. Ar-Larp is thus an upstream signal of tRNA trafficking that regulates cell cycle progression in response to environmental stresses.

## Results and discussion

### Progress and characterization of cell cycle arrest during *Artemia* diapause formation

**Fig. 1:**
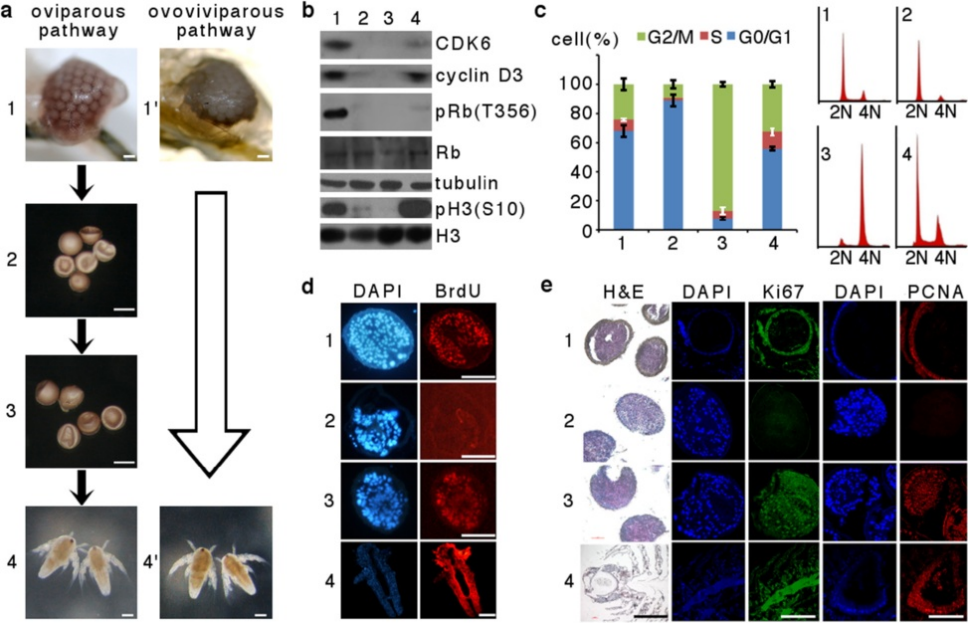
Progression and characterization of cell cycle arrest during *Artemia* diapause embryo formation and termination. **a** Developmental stages of *Artemia* during diapause formation (oviparous pathway) and direct development (ovoviviparous pathway). 1, Pre-diapause (early embryos); 2, diapause; 3, post-diapause; 4, nauplii; 1', early embryos; 4', nauplii. Scale bar = 1 mm. **b** Expression of the mitosis markers CDK6, cyclin D3, phosphorylated Rb (Thr356), and phosphorylated histone H3 (Ser10) at various stages of development. The lane numbers correspond to the developmental stages shown in (a). Histone H3 (H3) and α-tubulin were used as the loading controls for the nucleus and cytoplasm, respectively. **c** Analysis of the cell cycle phase during various stages of development. Flow cytometry analysis was performed with a fixed cell suspension stained with PE at each stage. The right panel shows the DNA content of cells in each stage during diapause formation. **d** 5-bromo-2’-deoxyuridine (BrdU) incorporation assay and **e** immunofluorescence of the proliferation markers Ki67 and proliferating cell nuclear antigen at each stage of *Artemia* during diapause formation. Scale bar = 500 μm

As a survival strategy, *Artemia* possesses two independent reproductive pathways that allow adaptation to widely fluctuating environments. Under unfavorable conditions, mature females produce and release encysted embryos that enter diapause, a state of obligate dormancy (oviparous pathway; Fig. 1a). Alternatively, under favorable conditions, they release swimming nauplius larvae directly (ovoviviparous pathway; Fig. 1a). To determine the cell division state in each developmental stage, Western blotting was performed to analyze the expression of the mitosis markers CDK6, cyclin D3, phosphorylated Rb at Thr356, and phosphorylated histone H3 at Ser10, all of which were strongly inhibited in the diapause and post-diapause stages (Fig. 1b). The results suggested that the cell cycle ceased during the diapause and post-diapause stages compared with the pre-diapause and larval stages, in which cell division was widespread.

To distinguish the cell cycle phases of diapause and post-diapause embryos, which are characterized as non-dividing cells, their DNA content was analyzed by flow cytometry. Analysis of the cell population distribution in diapause embryos revealed that more than 90 % of cells were in G0/G1 phase, whereas in post-diapause embryos, more than 85 % of cells were in G2/M phase, with very few cells in G0/G1 phase (Fig. 1c). These results were validated by the incorporation of 5-bromo-2’-deoxyuridine (BrdU). The lack of any BrdU signal in diapause embryos revealed that the cell cycle had been arrested before S phase; in contrast, the signal could be detected in post-diapause embryos that had progressed to G2/M phase during activation (Fig. 1d). Furthermore, the proliferation markers Ki67 and proliferating cell nuclear antigen were not detected in cells of diapause embryos, but were detected in cells of pre-diapause and post-diapause embryos and nauplii (Fig. 1e).

### Characterization of a La-related protein from *Artemia* during diapause formation

**Fig. 2:**
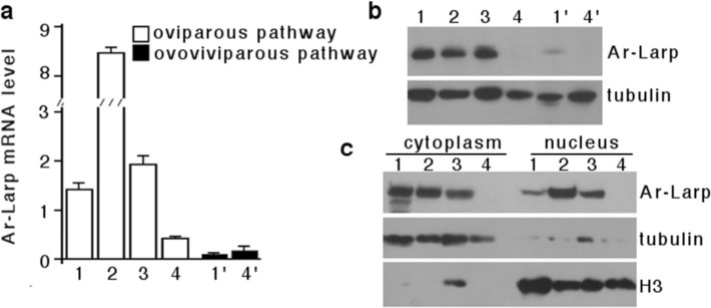
*Ar-Larp* gene expression in each developmental stage of *Artemia* in the oviparous and ovoviviparous pathways. **a** Real-time quantitative PCR analysis of Ar-Larp expression during each developmental stage (indicated in Fig. 1a). Error bars represent the mean ± SD of three independent experiments. **b** Western blot analysis of Ar-Larp during each developmental stage using α-tubulin as the loading control. **c** Subcellular location of Ar-Larp. Western blot analysis of Ar-Larp in the cytoplasm and nucleus during each developmental stage of the oviparous pathway. Histone H3 (H3) and α-tubulin served as the loading controls for the nuclear and cytoplasmic samples, respectively

During *Artemia* diapause formation, a gene encoding an RNA-binding protein is expressed specifically in oocytes in the *Artemia* ovisac [25]. In the current study, the expression of this gene, named *Ar-Larp*, was characterized by real-time PCR. This showed that the gene was expressed strongly in diapause-destined *Artemia*, but at a very low level in the directly developing pathway. In the former, *Ar-Larp* gene expression levels increased during diapause formation, reaching a maximum level in the encysted gastrula. *Ar-Larp* expression then decreased in post-diapause gastrula and disappeared in hatched nauplii (Fig. 2a).

The Ar-Larp cDNA was cloned and sequenced to reveal a 1215 bp region with a single open reading frame encoding a 404 amino acid (aa) protein (Additional file 1: Figure S1). The molecular mass and isoelectric point of the Ar-Larp protein were calculated as 46.81 kDa and 8.60, respectively. According to the structure analysis, Ar-Larp contains an La motif (aa position 9–88) and two RNA recognition motifs, RRM1 (aa position 101–170) and RRM2 (aa position 282–365), which are found in La and Larp proteins of many other species, ranging from trypanosomes to humans (Additional file 1: Figure S2). However, comparing the sequence with other proteins in the DDBJ/EMBL/GenBank database revealed that Ar-Larp has very low sequence identity to other known proteins (less than 20 %).

Using polyclonal antibodies specific to Ar-Larp, a band of about 47 kDa was identified in several stages of diapause-destined embryos, including oocytes and early, diapause, and post-diapause embryos, but not in hatched nauplii. In contrast, Ar-Larp was not detected in oocytes, embryos, or released nauplii of directly developing *Artemia* (Fig. 2b). In addition, Ar-Larp accumulated in the nuclei of diapause embryos, its level was decreased in the nuclei of post-diapause embryos, and it was undetectable in hatched nauplii (Fig. 2c). These results indicated that Ar-Larp is a diapause-specific protein. Furthermore, the nuclear accumulation and retention of Ar-Larp in diapause cysts of *Artemia* may have more important functions in cell cycle arrest during diapause embryo formation.

### Ar-Larp is required for diapause formation in *Artemia* and exhibits tRNA-binding activity

**Fig. 3:**
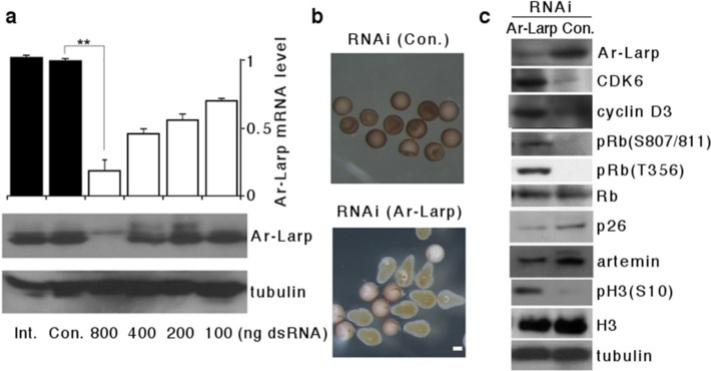
RNA interference (RNAi) of Ar-Larp in *Artemia*. **a** The expression levels of Ar-Larp mRNA were determined by real-time quantitative PCR after RNAi treatments. Int, intact; Ctrl, control treatment with 800 ng of GFP double stranded RNA (dsRNA). The proteins in diapause cysts were analyzed by Western blotting and tubulin was used as the loading control. **b** The phenotypes of RNAi-treated cysts injected with 800 ng of Ar-Larp dsRNA. Observation by the anatomical microscopy. Scale bar = 500 μm. Embryos treated with GFP dsRNA remained in diapause, whereas those treated with Ar-Larp dsRNA entered a pseudodiapause and developed into nauplii. **c** Western blot analysis of CDK6, cyclin D3, p26, Artemin, phosphorylated Rb (Thr356 and Ser807/811), and phosphorylated histone H3 (Ser10) after RNAi of Ar-Larp. Histone H3 (H3) and α-tubulin were used as the loading controls for the nucleus and cytoplasm, respectively

RNA interference (RNAi) was used to knockdown *Ar-Larp* gene expression to elucidate the function of Ar-Larp during the regulation of cell cycle arrest at the onset of *Artemia* diapause. Just before ovarian development, a double-stranded RNA (dsRNA), designed from the *Ar-Larp* cDNA sequence, was injected into the body cavity of adult oviparous *Artemia*. One day after the formation of diapause embryos (about 2 weeks post-injection), *Ar-Larp* mRNA levels decreased in proportion to increasing doses of the dsRNA (Fig. 3a). After injection of 800 ng of dsRNA, the *Ar-Larp* mRNA level was less than 15 % of that in the control, and Ar-Larp protein expression remained undetectable by immunoblot analysis. In contrast to the control, neither the cell cycle nor embryogenesis were arrested in gene-knockdown embryonic cells. This prevented diapause formation and produced pseudodiapause, in which the embryos continued to develop and hatch into nauplii (Fig. 3b). In gene-knockdown embryos, the restriction of cyclin D3 and CDK6 expression and the block in phosphorylation of Rb (at Thr356 and Ser807/811) and histone H3 (at Ser10) were removed (Fig. 3c). These results strongly suggested that the knockdown prevented the formation of a complete diapause state, resulting in a pseudodiapause embryo in which the cell cycle was not arrested, and the embryos continued to develop and hatch into nauplii.

To understand *Artemia* diapause formation, previous studies have focused on molecules associated with extreme environmental tolerance. For example, the small heat shock/α-crystallin protein p26, which acts as a molecular chaperone, is expressed in diapause cysts and is transported into nuclei during periods of environmental stress [26, 27]. In addition, a cyst-specific protein, artemin, has high thermal stability and functions as an RNA chaperone in the cytoplasm [28, 29]. In the current study, knockdown of the *Ar-Larp* gene prevented the formation of a complete diapause state and inhibited expression of p26 and artemin (Fig. 3c).

**Fig. 4:**
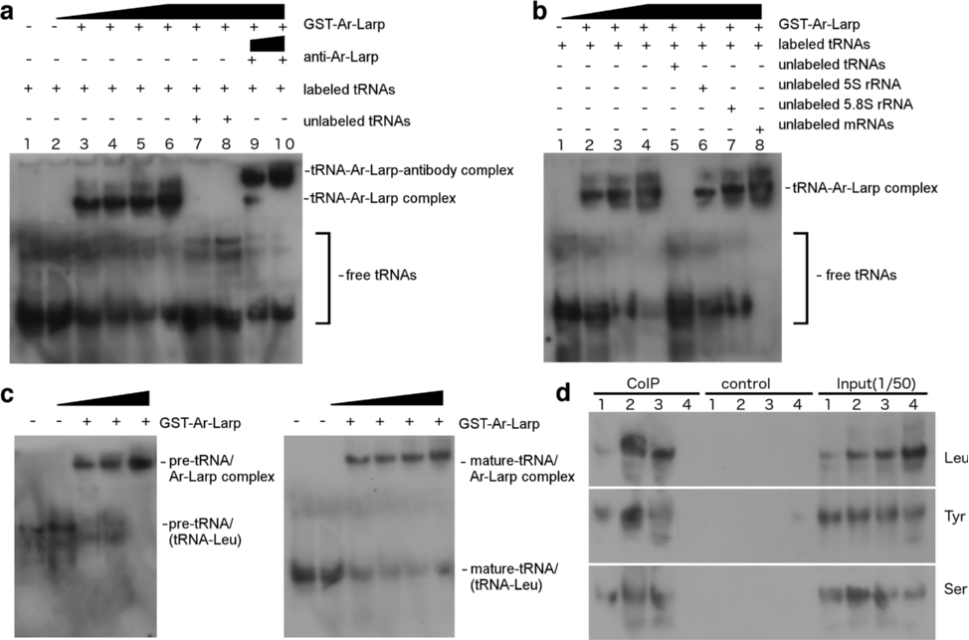
Analysis of the tRNA-binding activity of Ar-Larp. **a** Electrophoretic mobility shift assay (EMSA) analysis of the affinity between Ar-Larp and tRNAs. All tRNAs (25 μg per lane) were labeled with digoxigenin (DIG) using the DIG Oligonucleotide 3'-End Labeling Kit. Lanes 2–5 contain increasing amounts (0–1 μg) of GST-fused Ar-Larp. Ar-Larp-tRNA complexes were detected by Northern blotting (1 μg of GST was used as a control in lane 1). In lanes 7–8, excess tRNAs (250 and 500 μg, respectively) were added to the reaction system, which prohibited the binding of Ar-Larp to labeled tRNAs. In lanes 9–10, an anti-Ar-Larp antibody (0.5 and 2 μg, respectively) was added to the reaction system, and the super-shifted band of the Ar-Larp-anti-tRNA antibody complex was detected. **b** Competition EMSA analysis of the affinity between Ar-Larp and tRNAs and other RNAs. All tRNAs (25 μg per lane) were labeled with DIG using the DIG Oligonucleotide 3'-End Labeling Kit. Lanes 1–4 contain increasing amounts (0–1 μg) of GST-fused Ar-Larp. Ar-Larp-tRNA complexes were detected by Northern blotting. In lanes 5–8, excess RNAs (tRNA, 5.8 s rRNA, 5 s rRNA, and mRNAs, 500 μg each) were added to the reaction system as the competitors to prohibit the binding of Ar-Larp to labeled tRNAs. **c** EMSA analysis of the affinity between Ar-Larp and pre-tRNA and mature tRNA. Unlabeled tRNAs (25 μg per lane) were incubated with GST-Ar-Larp (0–1 μg). Ar-Larp-tRNA complexes were detected using probes specific to pre-tRNA (tRNA^Leu^, c-left panel) and mature tRNA (tRNA^Leu^, c-right panel) (1 μg of GST was used as a control in lane 1). **d** Detection of Ar-Larp-tRNA complexes by co-IP using an anti-Ar-Larp antibody and probes specific to tRNAs. Northern blot analysis of tRNAs from the pellets (co-immunoprecipitation) or total extract fractions (Input) of *Artemia* cells at each developmental stage (indicated in Fig. 1a, isotype antibody was used as a control)

Based on our structural analysis, Ar-Larp contains a La motif and two RRM motifs, suggesting that the protein is capable of recognizing and binding to RNAs. Here, we examined the interaction between recombinant Ar-Larp (GST-Ar-Larp) and tRNAs using an electrophoretic mobility shift assay (EMSA). The results showed that GST-Ar-Larp could form a complex with tRNA in vitro, and the complex was identified as a super-shifted band when an anti-Ar-Larp antibody was incubated together with Ar-Larp and tRNAs (Fig. 4a). To clarify the binding specificity of Ar-Larp to tRNA, several RNAs, including 5.8S rRNA, 5S rRNA, and mRNAs, were used as competitors of the binding. The result showed that none of these RNAs competed with the binding of Ar-Larp to tRNAs (Fig. 4b). Furthermore, the results of Northern blotting indicated that Ar-Larp could efficiently bind to both pre-tRNA and mature tRNA (Fig. 4c).

Both genetic and biochemical studies have revealed that a major role of La is to protect the 3’ end of nascent small RNAs from exonuclease digestion [30, 31]. Although less is known about the roles played by the La protein binding to mature tRNAs, there is a report demonstrating LARP7-mature tRNA interaction in vivo [32]. It should also be noted that *Artemia* oocytes are unusual cells, in that large quantities of proteins and RNAs are stored in preparation for early development. Thus, results obtained in *Artemia* oocytes may differ from other kinds of cells.

Next, co-immunoprecipitation (co-IP) was performed to investigate whether this interaction occurred in *Artemia*. Ar-Larp-tRNA complexes were identified in diapause embryos of *Artemia* and could be detected using several tRNA probes (Fig. 4d). However, the Ar-Larp-mRNA complex was not detected using probes specific for the mRNAs of tubulin, p26, and artemin, which are transcribed during diapause, indicating that Ar-Larp does not interact with mRNAs in *Artemia* (data not shown). Based on these results, we suggest that Ar-Larp controls cell cycle arrest in diapause by interacting with tRNAs in the nucleus.

### Ar-Larp localizes to the nucleus and induces cell cycle arrest in cancer cells

**Fig. 5:**
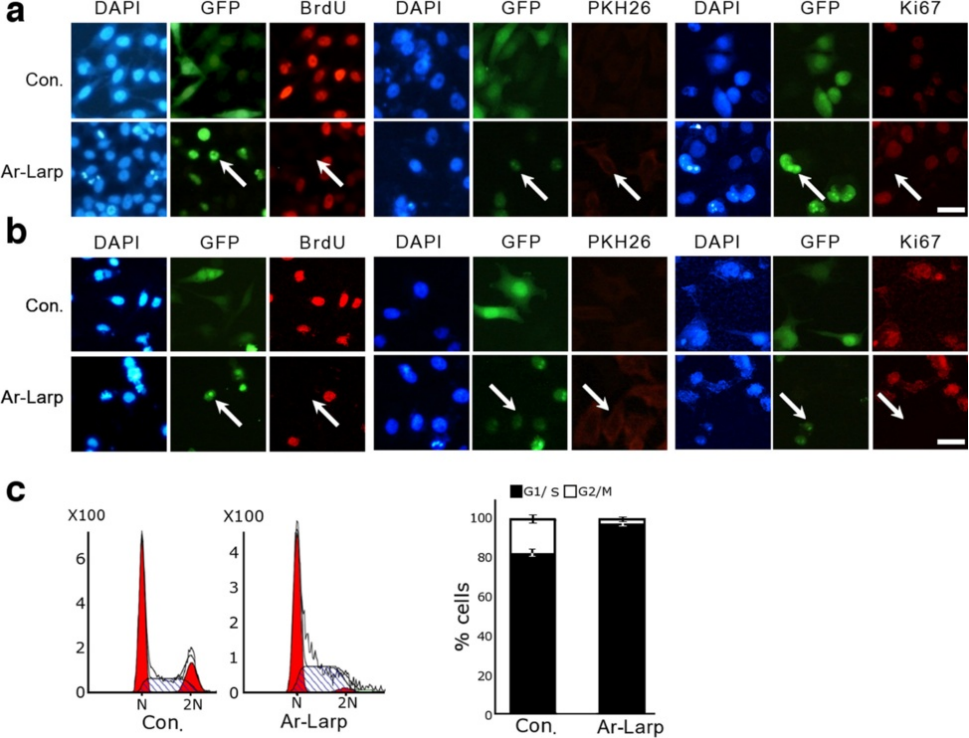
Ar-Larp overexpression results in cell cycle arrest in HeLa and MKN45 cells. Transient transfection of Ar-Larp in (**a**) HeLa and (**b**) MKN45 cells. Overexpressed green fluorescent protein (GFP) alone, green in control cells; overexpressed GFP-Ar-Larp, green in Ar-Larp-transfected cells. Mitosis was evaluated using the BrdU incorporation assay, the PKH26 label-retaining assay, and immunohistochemistry of Ki67. Cells overexpressing Ar-Larp (white arrows) lacked mitotic activity and remained in cell cycle arrest. **c** Flow cytometry analysis of the DNA content of HeLa cells using GFP (control) and GFP-*Ar-Larp* gene transfection. The bar graph shows the transition ratios from G1/S phase to G2/M phase in HeLa cells at 18 and 24 h after GFP-*Ar*-*Larp* gene transfection

To explore the function of Ar-Larp in the regulation of cell cycle arrest in systems other than *Artemia*, green fluorescent protein (GFP)-fused Ar-Larp was overexpressed in the human cancer cell lines HeLa (Fig. 5a) and MKN45 (Fig. 5b). Similar to the results obtained with the diapause embryos of *Artemia*, GFP-fused Ar-Larp localized to the nuclei in both of these cell lines (Fig. 5a,b). The results of the BrdU incorporation assay, the PKH26 label-retaining assay, and the expression of the proliferation marker Ki67 showed that, in contrast to control cells, cells overexpressing Ar-Larp had no mitotic activity (Fig. 5a,b). Flow cytometry analysis showed that most GFP-only transfected control cells progressed through S phase to G2/M phase, while GFP-*Ar-Larp*-transfected cells did not progress to G2/M phase and underwent cell cycle arrest (Fig. 5c). This result was also supported by a cell proliferation assay on soft agar, in which Ar-Larp clearly inhibited cell division and colony formation (Additional file 1: Figure S3). These results indicate that Ar-Larp induces cell cycle arrest in HeLa and MKN45 cells.

La-related proteins are conserved proteins with divergent structures and functions that interact with an extensive variety of cellular RNAs. Different La families may have evolved specialized activities in RNA metabolism [18]. Aberrant nuclear trafficking of La protein leads to the disordered processing of associated precursor tRNAs [31]. Although Ar-Larp has a low sequence identity to other known Larps, we found that it contains an La motif and two RRMs that are conserved in the Larp family (Additional file 1: Figure S4). To identify the Ar-Larp motifs that are important for its nuclear localization, its tRNA-binding, and its ability to regulate cell cycle arrest, six Ar-Larp mutants with various motif deletions were constructed and purified. The results of the tRNA-binding activity of each mutant indicated that both RRM1 and RRM2 participate in the tRNA-specific binding activity of Ar-Larp (Additional file 1: Figure S5). The La motif of Ar-Larp did not show tRNA-binding activity, although it had typical secondary and tertiary structures (Additional file 1: Figure S4). However, when this motif was aligned with other known La proteins, Phe residues at 23, 33, and 55 positions conserved in β-sheet 1 and 2 of the La motif in the proteins were changed to Leu, Val, and Cys residues in Ar-Larp. These conserved Phe residues are considered to be important for RNA recognition and tRNA-binding of the La motif in these proteins. Furthermore, the expression of the Ar-Larp mutants in HeLa cells suggested that the linker between RRM1 and RRM2 is associated with the retention of Ar-Larp in the nuclei of these cells (Additional file 1: Figure S6). Based on these results, six additional Ar-Larp mutants with various motif deletions were constructed and overexpressed in HeLa cells. BrdU incorporation was analyzed to detect the proliferation status. Mutants that had tRNA-binding activity without being retained in nuclei (Additional file 1: Figure S7a,b) or were retained in nuclei without tRNA-binding activity (Additional file 1: Figure S7e) could not induce cell quiescence. Only a combination of the nuclear retention (linker) and tRNA-binding (RRM1 and/or RRM2) regions could induce cell quiescence (Additional file 1: Figure S7c,d,f).

**Fig. 6:**
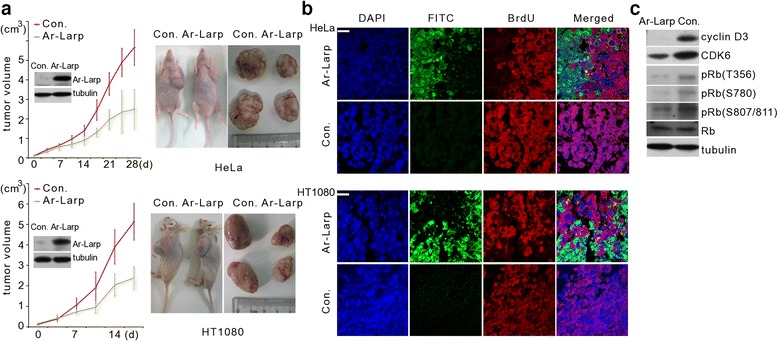
Ar-Larp inhibits mitosis of HeLa and HT1080 cells in mouse xenograft tumor models. **a** Female BALB/c nude mice were intratumorally treated with Ad-Ar-Larp or Ad-control after the formation of HeLa or HT1080 tumors. Tumor volumes and images are shown. The error bars represent the mean ± SD for five animals. Ar-Larp expression in the tumors was detected by Western blotting (insert). **b** Mitotic activity of cells in HeLa and HT1080 tumors examined using the BrdU incorporation assay after treatment with Ad-Ar-Larp or Ad-control. Blue, nuclei counterstained with DAPI. Green, Ar-Larp detected with a FITC-conjugated anti-Ar-Larp antibody. Red, incorporated BrdU detected with a rhodamine-conjugated anti-digoxigenin antibody. There is no mitotic activity in the green cells in the merged fields. Scale bar = 50 μm. **c** Expression of Ar-Larp and mitosis markers including CDK6, cyclin D3, and phosphorylated Rb (Thr356, Ser780, and Ser807/811). Tubulin was used as a loading control

To investigate whether Ar-Larp could induce cell quiescence in a mouse tumor model, an Ar-Larp adenovirus (Ad-Ar-Larp) was constructed and injected into HeLa and HT1080 human tumor xenograft model mice. Western blotting revealed the presence of Ar-Larp in tumor specimens, indicating that the protein was successfully expressed from the adenovirus construct in vivo (Fig. 6a insertion). Ar-Larp suppressed the growth of both HeLa and HT1080 tumors, representing 56 % and 55 %, respectively, of tumor formation in control mice (Fig. 6a). Importantly, BrdU labeling and detection revealed that all Ar-Larp-expressing cells in both HeLa and HT1080 tumors showed no mitotic activity compared with their controls (Fig. 6b). Further analysis indicated that expression of cyclin D3, CDK4/6, and phosphorylated Rb was strongly inhibited in HeLa tumors expressing Ar-Larp in contrast to the controls (Fig. 6c). Based on these results, we concluded that Ar-Larp is capable of inducing cell cycle arrest in tumors as well as in *Artemia* embryos.

### Ar-Larp controls cell cycle arrest by retrograde transport of tRNAs to the nucleus and its signaling pathways

**Fig. 7:**
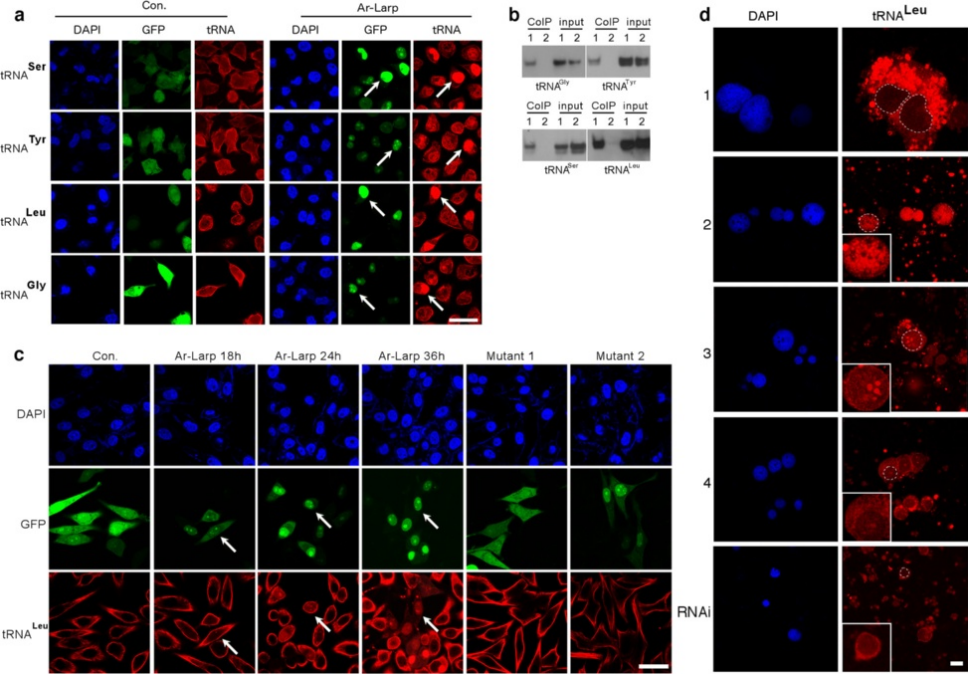
Ar-Larp retrogradely transports tRNAs from the cytoplasm to the nucleus, resulting in cell cycle arrest. **a** The tRNA distribution in HeLa overexpressing GFP-Ar-Larp (white arrows indicate typical cells) or GFP alone. tRNAs were detected by FISH using oligonucleotide probes complementary to tRNA sequences. Green, GFP-fused Ar-Larp and GFP alone. Red, tRNAs detected with a rhodamine-conjugated anti-digoxigenin (anti-DIG) antibody. Blue, nuclei counterstained with DAPI. Scale bar = 50 μm. **b** Detection of Ar-Larp-tRNA complexes by co-immunoprecipitation using an anti-Ar-Larp antibody and probes specific to the tRNAs. Northern blot analysis of tRNAs from the pellets (co-IP) or total extract fractions (Input) of HeLa cells overexpressing GFP-Ar-Larp. Lane 1, HeLa cells overexpressing GFP-Ar-Larp; lane 2, control cells overexpressing GFP alone. **c** Analysis of the retrograde transport of tRNA from the cytoplasm to the nuclei in Ar-Larp-overexpressing HeLa cells. HeLa cells were treated with ActD (5 μM) to block tRNA synthesis for 2 h after Ar-Larp was expressed for 8 h. The nuclear and cytoplasmic distributions of tRNA in HeLa cells were then detected by FISH using oligonucleotide probes (tRNA^Leu^) complementary to the tRNA sequences at 18, 24, and 36 h after Ar-Larp expression (white arrow indicates typical cells). Cells expressing GFP alone and two Ar-Larp mutants were used as controls. Mutant 1 lacked the domain between RRM1 and RRM2, and therefore was not retained in the nucleus. Mutant 2 could accumulate and be retained in the nucleus, but lacked the RNA-binding domain. Green, GFP-fused Ar-Larp and GFP alone. Red, tRNAs detected with a rhodamine-conjugated anti-DIG antibody. Blue, nuclei counterstained with DAPI. Scale bar = 50 μm. **d** The distribution of tRNAs in cells at each stage during diapause formation (the stages are indicated in Fig. 1a and in pseudodiapause cysts induced by *Ar*-*Larp* RNA interference. Dashed line circles indicate cells in each stage and pseudodiapause cysts. Scale bar = 50 μm

To address the function of Ar-Larp in cell cycle regulation, GFP-fused *Ar-Larp* was transfected into HeLa cells and tRNA trafficking was analyzed. Fluorescence in situ hybridization (FISH) using tRNA-specific probes was used to examine the distribution of tRNAs. The results indicated that tRNAs accumulated significantly in the nuclei of *Ar-Larp*-transfected HeLa cells compared to the controls (Fig. 7a). In addition, a co-IP assay was performed using an anti-Ar-Larp antibody to identify the tRNAs that accumulated in the nucleus. Immunoprecipitates were detected in cells transfected with the GFP-fused *Ar-Larp* gene using several tRNA probes (Fig. 7b). The results suggested that Ar-Larp induces cell cycle arrest by regulating tRNA trafficking.

To address whether the accumulation of tRNAs in the nucleus was due to the suppression of trafficking to the cytoplasm or retrograde transport from the cytoplasm, actinomycin D (ActD, 5 μM) was used to block de novo tRNA synthesis. In complete medium, tRNA could not be detected in the nuclei of control cells treated with ActD; however, tRNA accumulation in the nuclei was observed in Ar-Larp-overexpressing cells treated with ActD (Fig. 7c). During the nuclear transport of Ar-Larp (from 18 to 36 h after expression), the accumulation and retention of tRNAs was increased. The results indicated that tRNAs accumulated in the nuclei of Ar-Larp-overexpressing cells by moving retrogradely from the cytoplasm through the nuclear transport of Ar-Larp.

Although the regulation of post-transcriptional gene expression by tRNA nuclear accumulation remains an unproven possibility, evidence indicates that retrograde tRNA nuclear accumulation occurs in response to environmental stress. Previous studies reported that tRNA moves retrogradely from the cytoplasm to the nucleus in rat hepatoma and *S. cerevisiae* cells in response to aa deprivation [9, 10, 33, 34]. Thus, retrograde tRNA nuclear import may serve a regulatory role, separating tRNA from the translation machinery and resulting in the down-regulation of translation. However, the mechanism underlying tRNA nuclear import is unclear. In this report, our results indicate that Ar-Larp can function directly in retrograde tRNA nuclear import.

Based on the structural analysis, Ar-Larp from *Artemia* contains a La motif and two RRMs that are also found in La protein and LARP7. However, we found that Ar-Larp shows only 18 % and 17 % sequence identities to human La protein and LARP7, respectively (Additional file 1: Figure S8). Furthermore, human La protein and LARP7 did not show the activity of regulation in cell cycle arrest when they were overexpressed in HeLa cell line (Additional file 1: Figure S9). The previous report indicated that LARP7 inhibits breast cancer progression by binding to P-TEFb and 7SKsnRNA [35]. In this study, the complex of Ar-Larp binding with P-TEFb and 7SKsnRNA was not observed in Ar-Larp overexpressing HeLa cells by co-IP analysis and in vitro by EMSA analysis (Additional file 1: Figure S10). In addition, we did not find any homologue of Ar-Larp in HeLa cell by using anti-Ar-Larp antibody (Additional file 1: Figure S11). The results indicated that the structure and function of Ar-Larp is different to those of human La protein or LARP7 and the structure of molecule retrograded tRNA from cytoplasm to nucleus leading to cell cycle arrest in human maybe different with that of Ar-Larp.

Furthermore, we compared the effect of Ar-Larp and protein synthesis inhibitors, such as puromycin, cycloheximide, and fusidic acid, which block protein synthesis in the elongation or translocation steps. Unlike cell cycle arrest induced by Ar-Larp, tRNA retrograde movement from the cytoplasm to the nucleus was not observed in MKN45 cells treated with these inhibitors (Additional file 1: Figure S12).

The tRNA distribution in cells of each stage during diapause formation and in pseudodiapause cysts induced by *Ar-Larp* RNAi in *Artemia* was identified by FISH analysis using a tRNA^Leu^ probe (Fig. 7d). The result showed that the distribution of tRNAs coincided with the expression and distribution of Ar-Larp. In diapause cyst cells, tRNAs accumulated in nuclei; however, in pseudo diapause cyst cells induced by Ar-Larp RNAi (as shown in Fig. 3b), the accumulation of tRNAs in nuclei disappeared simultaneously (Fig. 7d). Based on the results obtained in this study, we propose that Ar-Larp regulates cell quiescence through its involvement in the retrograde nuclear accumulation of cytoplasmic tRNAs.

In our previous studies, we found that mitogen-activated protein kinase-ERK-ribosomal S6 kinase 1 is inhibited and H3K56ac accumulates in diapause embryos [23, 36]. To clarify the signaling pathways linking tRNA trafficking to the cell cycle checkpoint, the pathways concerned were analyzed by Western blotting. Of the various processes and pathways involved, the level of H3K56ac remained high, while the phosphorylation of ERK1/2 and Akt was strongly inhibited. In addition, the activation of FoxO3a was also inhibited in HeLa cells after *Ar-Larp* gene transfection (Additional file 1: Figure S13). However, Ar-Larp did not appear to have any effect on the molecules of the other tested signaling pathways (Additional file 1: Figure S14).

To confirm that Ar-Larp was responsible for activating certain signaling pathways in *Artemia*, RNAi was performed as described above. After knockdown of the *Ar-Larp* gene, the level of H3K56ac was reduced and phosphorylation of ERK1/2 and Akt was increased (Additional file 1: Figure S15). Based on these findings, we propose the molecular mechanism underlying Ar-Larp-induced cell cycle arrest, as identified in this study. Ar-Larp retrogradely transports tRNAs to the nucleus, followed by the up-regulation of H3K56ac, and the down-regulation of ERK1/2 and Akt phosphorylation and inhibition of FoxO3a activation. Consequently, the cyclin D3/CDK6 complex is inactivated, while Rb is maintained in an active state to perform its function as a tumor suppressor by inhibiting cell cycle progression.

## Conclusions

The regulation of the cell cycle involves processes that are crucial to survival, including the detection and repair of genetic damage and the prevention of uncontrolled cell division. Encysted gastrula in diapause released from *Artemia* provides an excellent model system for studying cell biology and the biochemical adaptation to extreme environments. In this study, we used *Artemia* as a peculiar model for cell cycle studies, and describe in detail how the *Artemia* RNA-binding protein Ar-Larp controls the onset of *Artemia* diapause, induces cell cycle arrest at G1/S phase in human cancer cells, and suppresses tumor growth in a xenograft mouse model. From *Artemia*, we have learned considerably about how cysts protect the embryos from extremely harsh environments, utilizing the capacity to maintain the embryos in a state of cell cycle arrest for long periods of time. Unlike protein inhibitors, Ar-Larp controls tRNA nucleocytoplasmic trafficking, transducing the signal to the cell cycle checkpoints, including H3K56ac, ERK, and Akt, which suppresses cell division. In this sense, Ar-Larp is an extraordinary protein and will be extremely useful in providing further insights into the regulation of cell division. Based on the unique structure and biological function of the Ar-Larp molecule, Ar-Larp and its related processes should help to provide novel approaches to non-specific gene therapies for cancer treatment.

## Methods

### Animals and ethics statement

Diapause-destined *Artemia* (*Artemia parthenogenetica*) were raised in 8 % (w/v) artificial seawater (Blue Starfish) at 25 °C on a 4L:20D cycle, while the directly developing *Artemia* were reared in 4 % (w/v) artificial seawater on a 16L:8D cycle. Chlorella powder (Fuqing King Dnarmsa Spirulina Co., Ltd.) was supplied as brine shrimp food. BALB/c nude mice aged 6–8 weeks (Institution of Animal Research, Shanghai, China) were used as xenograft recipients for cancer cells. Animal experiments were approved and performed in accordance with the institutional guidelines for animal care of the animal ethics committee of Zhejiang University.

### Cell culture and transfection

HeLa, HT1080, and MKN45 cells (ATCC, VA, USA) were cultured at 37 °C in 5 % CO_2_ in DMEM, RPMI-1640, and EMEM (Genom, China), respectively, supplemented with 10 % fetal bovine serum (Gibco, MT, USA). Cell transfections were performed with Lipofectamine 2000 (Invitrogen, CA, USA) as recommended by the manufacturer.

### Western blotting and antibodies

Ar-Larp protein with His-tag was expressed in *Escherichia coli* BL21 (DE3) and purified using Ni-NTA Agarose (Qiagen, Germany). The anti-Ar-Larp antibody was raised in rabbits (HuaAn, China). For nuclear and cytoplasmic fractions, the extracts were prepared using NPE Nuclear and CPE Cytoplasmic Extraction Reagents (Bioteke, China) following the manufacturer’s instructions. Proteins were extracted from each tissue using protein loading buffer (0.2 mM dithiothreitol, 4 % (w/v) sodium dodecyl sulfate (SDS), 0.2 % (w/v) bromophenol blue, and 20 % (v/v) glycerol prepared in 0.1 mM Tris-HCl, pH 6.8). Fifty micrograms of protein of each sample were separated on 12.5 % SDS-PAGE gels and transferred to PVDF membranes (Millipore, MA, USA). The membranes were incubated with antibodies overnight at 4 °C and detected using the BM Chemiluminescence Western Blotting Kit (Roche, Germany) following the manufacturer’s instructions.

### Flow cytometry analysis

Fluorescence-activated cell sorting was performed as previously described [23]. Data analysis was accomplished with Cell Quest™ software, version 3.1 (BD Biosciences, NJ, USA). All data are presented as the mean ± SD. All statistical analyses were performed using the two-tailed paired Student’s *t*-test. The differences were considered statistically significant at *P* <0.01.

### BrdU incorporation assay

*In vivo* BrdU labeling was performed according to the manufacturer’s instructions (Sigma, MO, USA). Cells were incubated with 12 mM BrdU (Sigma) for an additional 24 h after transfection. The incorporation of BrdU into the genome was detected with a mouse anti-BrdU primary antibody (Sigma) followed by a secondary goat anti-mouse antibody conjugated to rhodamine (HuaAn). Wide-field fluorescence microscopy was used to obtain images.

### Quantitative real-time PCR

Total RNA was isolated using TRIzol reagent (Invitrogen). cDNA was synthesized from 2 μg of total RNA using a SuperScript™/First-Strand cDNA Synthesis kit (Invitrogen) with Oligo(dT) primers. cDNA was diluted 10-fold and combined with SYBR Green Supermix (TaKaRa, Japan) for quantitative PCR. The relative abundance of mRNA was normalized to that of α-tubulin. The results are reported as the mean ± SD of three independent experiments. Comparisons between groups for statistical significance were performed with a two-tailed paired Student’s *t*-test.

### RNAi of the *Ar-Larp* gene

Recombinant plasmids expressing Ar-Larp dsRNAs were constructed and the dsRNAs were induced, expressed, and purified as described previously [23]. Ar-Larp dsRNA (800 ng) was injected into the reproductive segment of *Artemia* using an UltraMicroPump II equipped with a Micro4™ MicroSyringe Pump (World Precision Instruments, FL, USA). Similarly, GFP dsRNA was injected as the negative control.

### EMSA

For the EMSA, yeast tRNAs (Ambion, USA) were first labeled with the digoxigenin (DIG) Oligonucleotide 3'-End Labeling Kit (Roche, USA) and then incubated with various concentrations of a GST-Ar-Larp fusion protein (0–1 μg) or GST alone (1 μg) in 20 μL of RNA-binding buffer (Tris (pH7.5) 0.1 M, KCl 0.5 M, dithiothreitol 10 mM, with diisodecyl glutarate) at room temperature for 30 min. The reaction mixtures were electrophoresed on 12.5 % non-denaturing polyacrylamide gels in Tris/Borate/EDTA buffer, and detected using the Northern Blotting Kit (Roche, Germany) following the manufacturer’s instructions. For the super-shift assay, an anti-Ar-Larp antibody (0.5–1 μg) was incubated simultaneously in the EMSA system. For competition EMSA experiments, several excess competitors (10-fold unlabeled tRNA, 5.8*S* rRNA, 5*S* RNA, and mRNAs) were added to the EMSA system.

### Co-IP

Crude protein extracts were prepared in the presence of proteinase inhibitors (Sangon, China) and the RiboLock™ Ribonuclease Inhibitor (MBI, CT, USA), and were incubated with an anti-Ar-Larp antibody (HuaAn) at 4 °C overnight, followed by the addition of Protein A Dynabeads (Invitrogen) for 3 h at 4 °C. The beads were washed with extraction buffer twice, and tRNAs were purified using TRIzol reagent (Invitrogen). RNA was loaded onto 8 % urea denaturing polyacrylamide gels prior to hybridization with tRNA oligonucleotide probes at 39 °C.

### Mouse xenograft model

A recombinant adenovirus expressing Ar-Larp (Ad-Ar-Larp) was constructed and purified using the Adenovirus Dual Expression Kit (TaKaRa) following the manufacturer’s instructions. Tumor-bearing BALB/c nude mice were injected with Ad-Ar-Larp or a control adenovirus (Ad-control) twice a week. Tumor volumes were determined by measuring tumors in two dimensions as described previously [37]. Four weeks later, the mice were injected with BrdU (50 mg/kg) and ten hours later, the mice were sacrificed and the tumors were harvested and paraffin-embedded. BrdU incorporation was detected as described above.

### FISH

tRNA FISH was performed as described previously [38]. To block de novo tRNA synthesis, cells were treated with 5 μM ActD (Sigma) for 2 h and then cultured in complete medium. Cells were fixed and incubated with 3’ end DIG-labeled tRNA oligonucleotide probes for hybridization at 39 °C. A rhodamine-conjugated anti-DIG Fab fragment (Roche, Germany) was used to determine the tRNA abundance. The tRNA oligonucleotide probes are listed in Additional file 2: Supplemental Materials and Methods.

### Availability of data and materials

Supporting information and additional methods can be found in Additional files 1, 2, and 3.

## Additional files

Additional file 1: Figure S1. Nucleotide sequence of Ar-Larp cDNA and its deduced amino acid sequences. **Figure S2.** Sequence alignment of the La (LAM), RRM1 and RRM2 motifs of Ar-Larp with the LARP7 protein family. **Figure S3.** Proliferation of HeLa cells overexpressing Ar-Larp. **Figure S4.** Structural predictions for the Ar-Larp. **Figure S5.** The affinity of Ar-Larp mutants for tRNA was analyzed by electrophoretic mobility shift assay (EMSA). **Figure S6.** Subcellular locations of six Ar-Larp mutants. **Figure S7.** Analysis of mitosis of HeLa cells overexpressing Ar-Larp mutants by BrdU-incorporation assay. **Figure S8.** Sequence alignment of Ar-Larp with human La protein and LARP7 protein. **Figure S9.** Analysis of mitosis of HeLa cells overexpressing human La protein and LARP7 by BrdU-incorporation assay. **Figure S10.** The affinity of Ar-Larp to 7SKsnRNA was analyzed by co-immunoprecipitation and EMSA. **Figure S11.** Proteins in HeLa cells were detected by anti-Ar-Larp antibody. **Figure S12.** The comparison of tRNA distribution of MKN45 after Ar-Larp overexpression and treated with general protein synthesis inhibitors. **Figure S13.** Analysis of signaling pathways in Ar-Larp-induced cell cycle arrest. **Figure S14.** Western blot analysis of cell cycle regulation related signaling pathways in HeLa cells 24, 36 and 48 h after GFP-fused Ar-Larp (GFP-Ar-Larp) or GFP only (Control) gene transfection. **Figure S15.** Western blot analysis of pAkt (T308), pERK1/2 (T202/Y204) and H3K56ac in diapause embryos of *Artemia* after Ar-Larp RNA interference treatment. (PDF 1459 kb) Additional file 2:
**Supplementary methods.** (DOC 65 kb) Additional file 3: Table S1. Primes used in this study. **Table S2.** Sequences obtained in this study. **Table S3.** Oligonucleotides used in this study. (DOC 74 kb)
