# Supplementary material for: An La-related protein controls cell cycle arrest by nuclear retrograde transport of tRNAs during diapause formation in Artemia
Source: BMC Biol. 2016 Mar 3;14:16. doi: 10.1186/s12915-016-0239-4 (PMC4778291; doi:10.1186/s12915-016-0239-4)
Supplement: Additional file 1: Figure S1. — Nucleotide sequence of Ar-Larp cDNA and its deduced amino acid sequences. Figure S2. Sequence alignment of the La (LAM), RRM1 and RRM2 motifs of Ar-Larp with the LARP7 protein family. Figure S3. Proliferation of HeLa cells overexpressing Ar-Larp. Figure S4. Structural predictions for the Ar-Larp. Figure S5. The affinity of Ar-Larp mutants for tRNA was analyzed by electrophoretic mobility shift assay (EMSA). Figure S6. Subcellular locations of six Ar-Larp mutants. Figure S7. Analysis of mitosis of HeLa cells overexpressing Ar-Larp mutants by BrdU-incorporation assay. Figure S8. Sequence alignment of Ar-Larp with human La protein and LARP7 protein. Figure S9. Analysis of mitosis of HeLa cells overexpressing human La protein and LARP7 by BrdU-incorporation assay. Figure S10. The affinity of Ar-Larp to 7SKsnRNA was analyzed by co-immunoprecipitation and EMSA. Figure S11. Proteins in HeLa cells were detected by anti-Ar-Larp antibody. Figure S12. The comparison of tRNA distribution of MKN45 after Ar-Larp overexpression and treated with general protein synthesis inhibitors. Figure S13. Analysis of signaling pathways in Ar-Larp-induced cell cycle arrest. Figure S14. Western blot analysis of cell cycle regulation related signaling pathways in HeLa cells 24, 36 and 48 h after GFP-fused Ar-Larp (GFP-Ar-Larp) or GFP only (Control) gene transfection. Figure S15. Western blot analysis of pAkt (T308), pERK1/2 (T202/Y204) and H3K56ac in diapause embryos of Artemia after Ar-Larp RNA interference treatment. (PDF 1459 kb) [file 12915_2016_239_MOESM1_ESM.pdf]

Supplementary File 1: Supplementary Figures and Figure Legends

An La-related Protein Controls Cell Cycle Arrest by Nuclear Retrograde Transport of tRNAs during Diapause Formation in *Artemia*

Dian-Fu Chen[1,2], Cheng Lin[1], Hong-Liang Wang[1], Li Zhang[1], Li Dai[1], Sheng-Nan Jia[1], Rong Zhou[3], Ran Li[4], Jin-Shu Yang[1], Fan Yang[1], James S Clegg[5] , Hiromichi Nagasawa[1, 6] and Wei-Jun Yang[1,*]

[1] College of Life Sciences, Zhejiang University, Hangzhou 310058, People's Republic of China

[2] Key Laboratory of Conservation Biology for Endangered Wildlife of the Ministry of Education, Zhejiang University, Hangzhou 310058, People's Republic of China

[3] Key Laboratory of Protein Chemistry and Developmental Biology of the State Education Ministry of China, College of Life Sciences, Hunan Normal University, Changsha, 410018, People's Republic of China

[4] Tianjin Key Laboratory of Animal and Plant Resistance, College of Life Sciences, Tianjin Normal University, Tianjin 300387, People's Republic of China

[5] Section of Molecular and Cellular Biology and Bodega Marine Laboratory, University of California, Davis, Bodega Bay, California 94923, USA

[6] Department of Biological Chemistry, The University of Tokyo, Yayoi, Bunkyo, Tokyo 113-8657, Japan

[*] Email: w_jyang@ zju.edu.cn.

Supplementary Figures

```
   1 GGCACGAGGGCAAATGCGAGTTTCCTTACAAGAAGAAAAAGAAATGGCAGAAAATATCCAACATGATGAACGCATACTTCGAAAAGTTAG
   1                                              M  A  E  N  I  Q  H  D  E  R  I  L  R  K  V  R
  91 GAAGCAAATTGAATTTTACTTAAGTGATGCAAACTTGTCTAAAGACCGGGTAACTTGCTGTACTTACTCTGAGGCCATCTCAAGTGGAGG
  17  K  Q  I  E  F  Y  L  S  D  A  N  L  S  K  D  R  V  T  C  C  T  Y  S  E  A  I  S  S  G  G
 181 TATTCCCGCAGATTTCTTCTTGAAATGCAATAGAGTAAAAGAACGCATTACAACAGTTGCTGAAATTGAAGAAGCGCTGAAGACATCTAA
  47  I  P  A  D  F  F  L  K  C  N  R  V  K  E  R  I  T  T  V  A  E  I  E  E  A  L  K  T  S  K
 271 ATATTTGCAATTTGCAGATGGAAAAGTCTCCAGAAAATTTCCTTTTCAACCTCGAACAAACCAGGATAGATGCACCATTTATGTGGAAAA
  77  Y  L  Q  F  A  D  G  K  V  S  R  K  F  P  F  Q  P  R  T  N  Q  D  R  C  T  I  Y  V  E  N
 361 TATTCCCACGTATGCAACGCAAGAGAAAGTTCGTAAATGGTTCCGACCTTACGGAAAGGTAGTTTATGTCTCCCTTCCTAAATCAAAAGT
 107  I  P  T  Y  A  T  Q  E  K  V  R  K  W  F  R  P  Y  G  K  V  V  Y  V  S  L  P  K  S  K  V
 451 AGGGACAATTAAAGGATATGCTTTTGTTGAGTTCAATACAGAAGAAGAAGCAGAAATCTGCATGTTATCCTATCAAGAATCTGGGAGATA
 137  G  T  I  K  G  Y  A  F  V  E  F  N  T  E  E  E  A  E  I  C  M  L  S  Y  Q  E  S  G  R  Y
 541 TATTGAAAACCGTGACCCAGCGGAGCTGTTATCAGTTAAAACGTTTGAAGGATTTGAAGATGCTGAGGTTGAAACTGAAACAGCGGAACC
 167  I  E  N  R  D  P  A  E  L  L  S  V  K  T  F  E  G  F  E  D  A  E  V  E  T  E  T  A  E  P
 631 TGAATATCAGCCCTTTGAGAGCGCTGAAGAGGCCCATCCTCAGGAAGTCACAAAAGAGGATGTCACCACTCAGTTTAGGATTCTTAGTAG
 197  E  Y  Q  P  F  E  S  A  E  E  A  H  P  Q  E  V  T  K  E  D  V  T  T  Q  F  R  I  L  S  R
 721 GAACGATTGGAAAAAGGAGAGAAACAAGTACCTGAATAACTGGAGGAAAGAAGGAAAGGCACGTAGGCAAGAAATTTGGAGGCAGCAGAT
 227  N  D  W  K  K  E  R  N  K  Y  L  N  N  W  R  K  E  G  K  A  R  R  Q  E  I  W  R  Q  Q  M
 811 GAAAATTAACCGAAGGAGGAAAATAGCCGAGGAGGAAAAAGCTTTACAGCCTAAAACGGAACTAAACTTCCAAAGCGGCTTGATTGTCAA
 257  K  I  N  R  R  R  K  I  A  E  E  E  K  A  L  Q  P  K  T  E  L  N  F  Q  S  G  L  I  V  K
 901 GATTTCTTTGAAGGAAGAAATTCATGATCCAAAATCTATAAAGGAACAATTAAAAGCTATTGGCGGCATAAAATATGTTGAAGCCATGCA
 287  I  S  L  K  E  E  I  H  D  P  K  S  I  K  E  Q  L  K  A  I  G  G  I  K  Y  V  E  A  M  Q
 991 AGGTGCAGTGGAAGCAATTGTCCGTACAGAAAGCCCAGATGTGGCCAGCTCACTTATCAAAAATCCATTAGGAAAGGGTGAAGTCCTTAC
 317  G  A  V  E  A  I  V  R  T  E  S  P  D  V  A  S  S  L  I  K  N  P  L  G  K  G  E  V  L  T
1081 TGGTGCTGAGGAACTAGAATATTGGTTTAAAATACTTTCAGGCAAGGAAGCGAAGCTTTCGGCATCAAAAGAAGTCAATAAAGTAAAGGT
 347  G  A  E  E  L  E  Y  W  F  K  I  L  S  G  K  E  A  K  L  S  A  S  K  E  V  N  K  V  K  V
1171 GAAGAAGAAGAAGAAAAAGGCTCAGCATATTCGGTTTGATGATGATGAAAATACAAAATCGGAAATAGAAACAACAGACGTGATTTGATA
 377  K  K  K  K  K  K  A  Q  H  I  R  F  D  D  D  E  N  T  K  S  E  I  E  T  T  D  V  I  *
1261 AGCTGTTTTCCGAAAAGCGTAGTTTATTGACGTGCATTATTTTTTTATGTATTTTACTTTTTTACTTTTTACAATATTTATTTTTTACTT
1351 TCTTGTAAGATTACATTTATACGTCAATACATTACATGATTAATAGAAAAAAAAAAAAAAAAAAAAAAAAAAAAAA
```

Fig. S1 Nucleotide sequence of Ar-Larp cDNA and its deduced amino acid sequences. The nucleotide and amino acid residue numbers are indicated on the left. The start (ATG) and stop (TGA) codons are indicated by shading and underlining, respectively. The asterisk denotes amino acid termination. The putative polyadenylation signal (AATACA) is boxed.

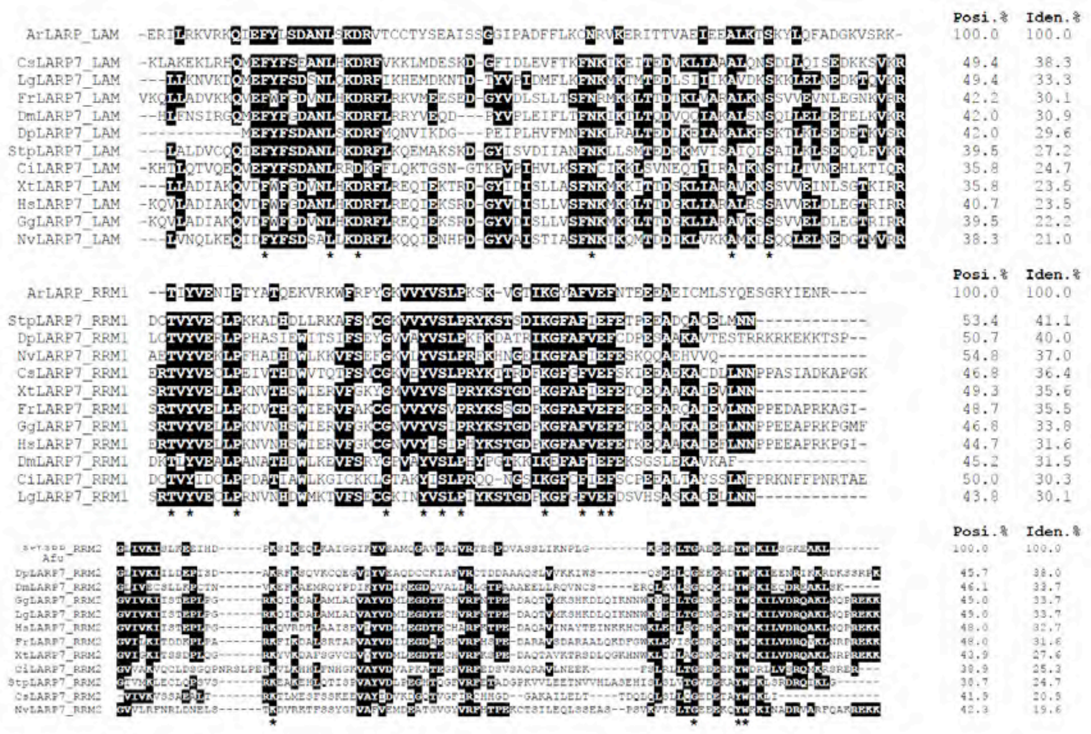

Fig. S2 Sequence alignment of the La (LAM), RRM1 and RRM2 motifs of Ar-Larp with the LARP7 protein family. The percentage positive and identical matches with Ar-Larp are indicated on the right. Identical amino acid residues are labeled with asterisks (*). Species abbreviations: Am: Apis mellifera; Ci: Ciona intestinalis; Cs: Capitella sp.; Dm: Drosophila melanogaster; Dp: Daphnia pulex; Fr: Fugu rubripes; Gg: Gallus gallus; Hs: Homo sapiens; Lg: Lottia gigantae; Nv: Nasonia vitripennis; Stp: Strongylocentrotus purpuratus; and Xt: Xenopus tropicalis.

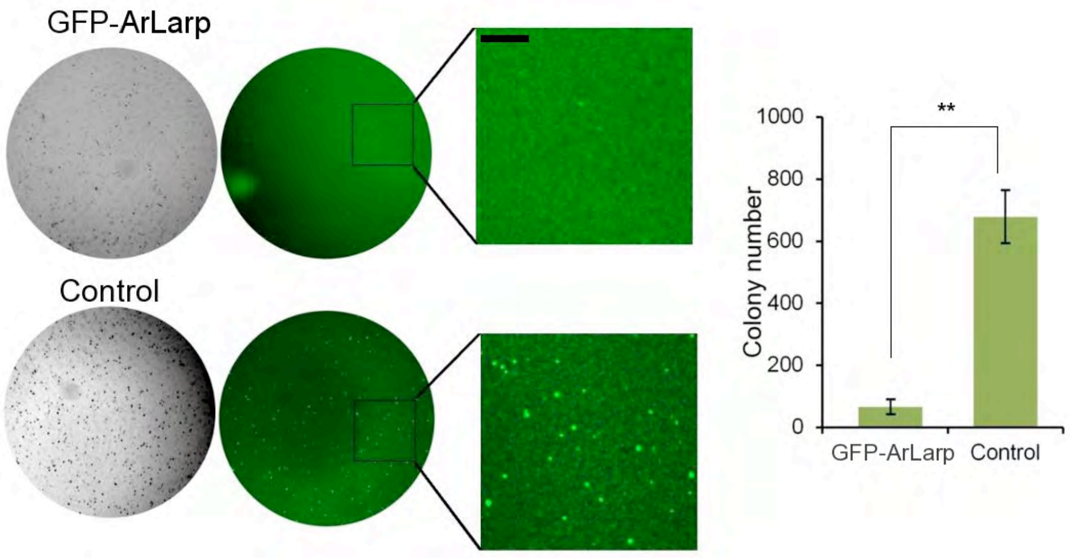

Fig. S3 Proliferation of HeLa cells overexpressing Ar-Larp. HeLa cells overexpressing GFP-Ar-Larp (Ar-Larp) or GFP alone (Con.) were grown in soft agar for 7 d. Representative colonies were photographed using a wide-field fluorescence microscope. The colonies in each soft agar assay were counted and are shown as histograms. The error bars represent the mean±S.D. from three independent experiments. **$P<0.01$.

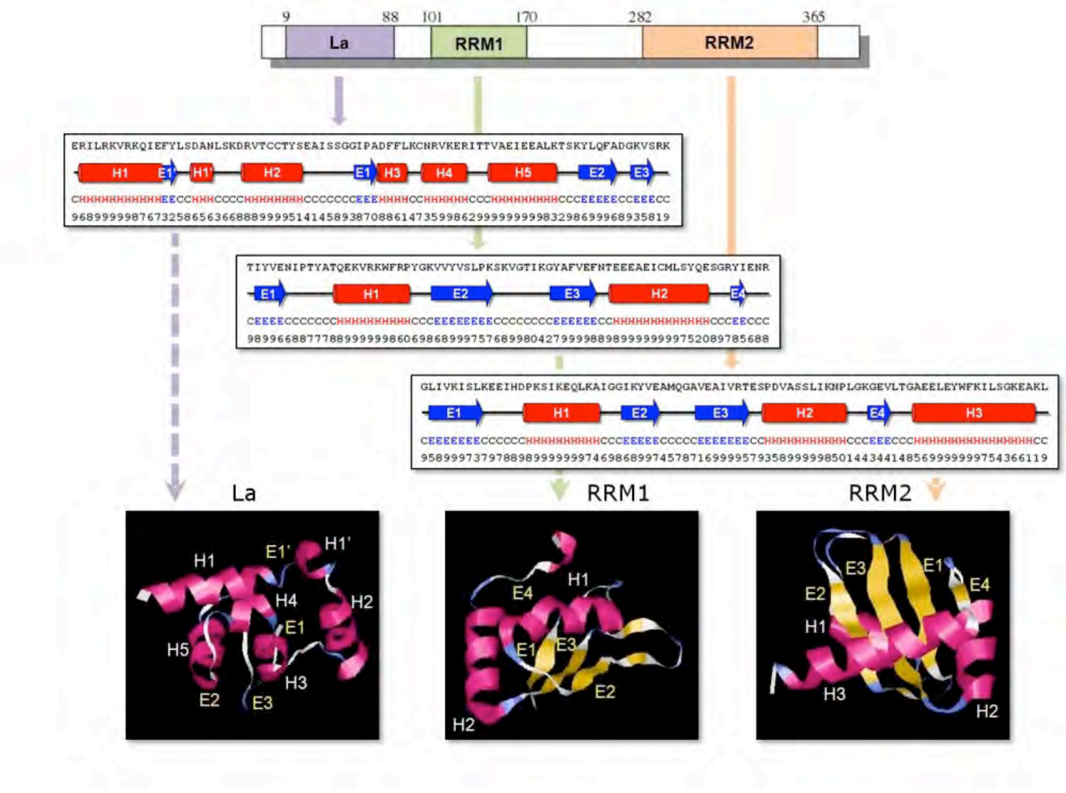

Fig. S4 Structural predictions for the Ar-Larp. Three putative conserved domains (La, RRM1, and RRM2) were detected using the Basic Local Alignment Tool (http://blast.ncbi.nlm.nih.gov/Blast.cgi). The secondary and tertiary structures of the conserved motifs were predicted using the PSIPRED and I-TASSER prediction packages, respectively. The numbers under the secondary structure units indicate confidence scores calculated from the PSIPRED results. Red boxes and blue arrows represent putative helixes and strands, respectively. The tertiary structures were visualized using RasMol. H: helix; E: strand; C: coil.

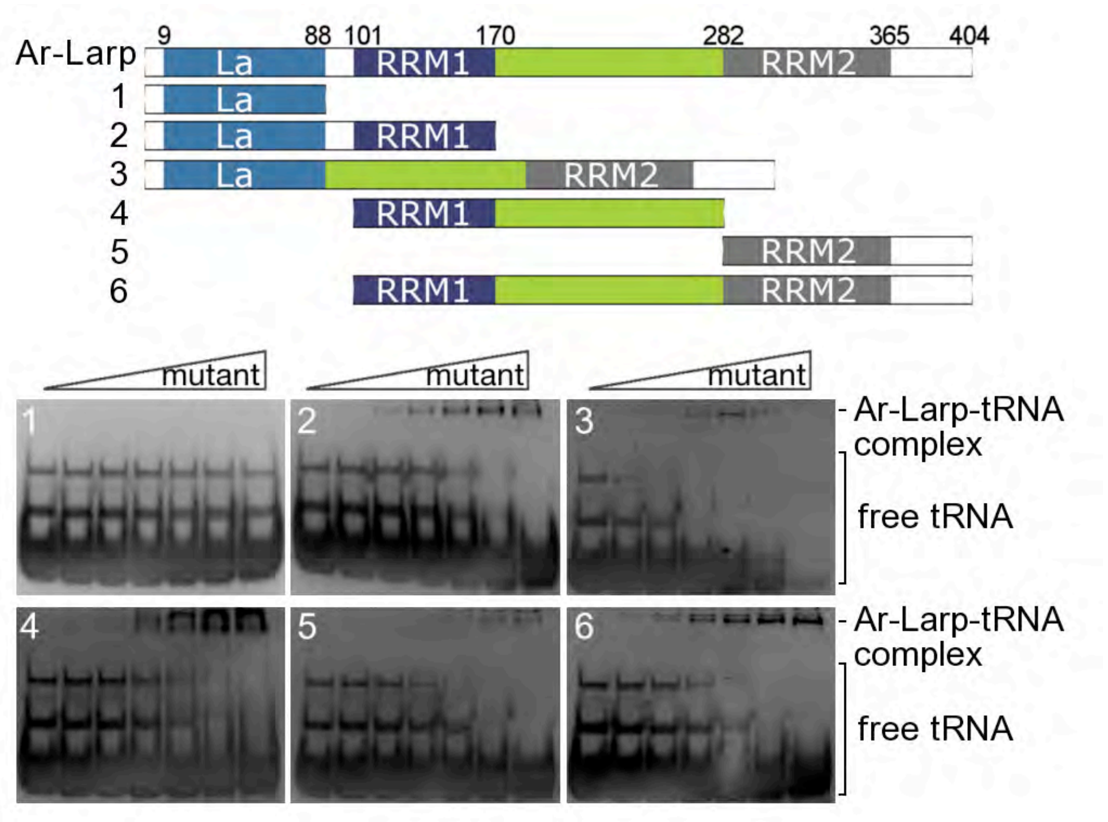

Fig. S5 The affinity of Ar-Larp mutants (1–6, as shown in the schematic representation above) for tRNA was analyzed by EMSA. The lanes contain GST-fused Ar-Larp mutants in increasing amounts from 0 to 10 μg. Ar-Larp mutant-tRNA complexes were detected with direct GoldView staining.

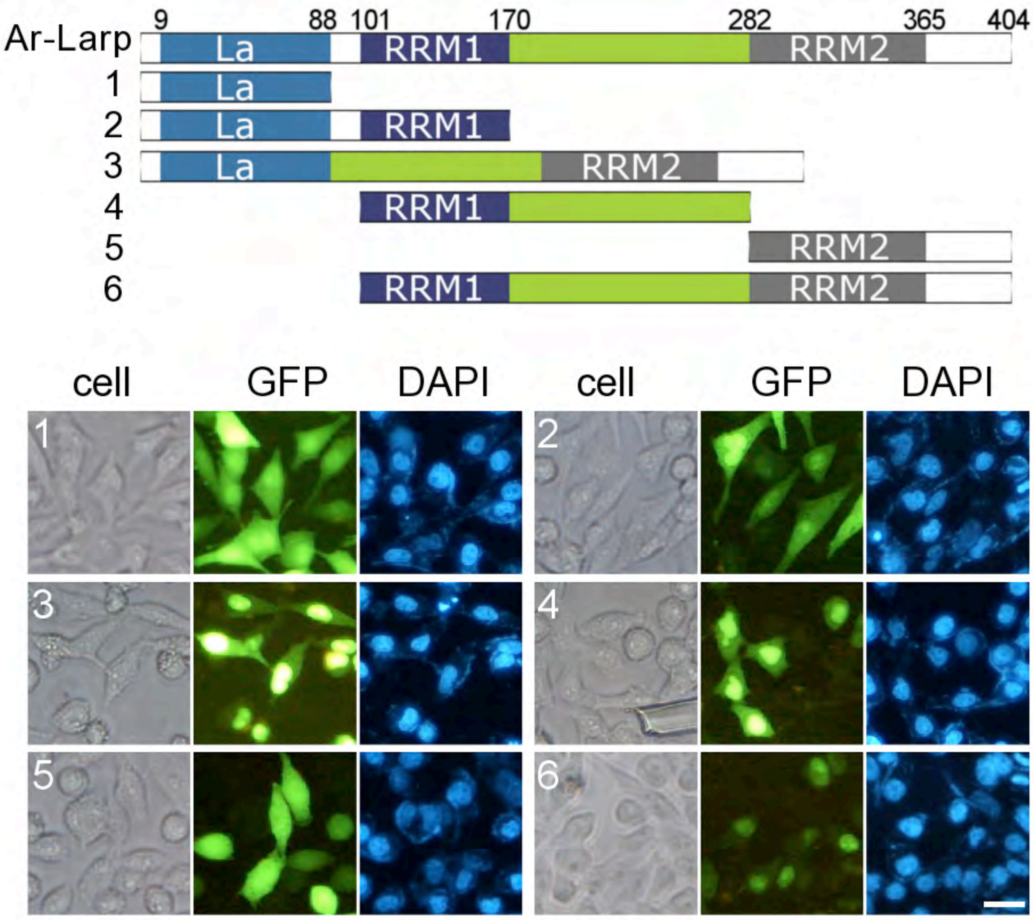

Fig. S6 Subcellular locations of six Ar-Larp mutants (1–6, as shown in the schematic representation above) were viewed using a fluorescence microscope 24 h after GFP-Ar-Larp transfection. Green, GFP-fused Ar-Larp mutants; Blue, nuclei counterstained with DAPI. Scale bar = 100μm.

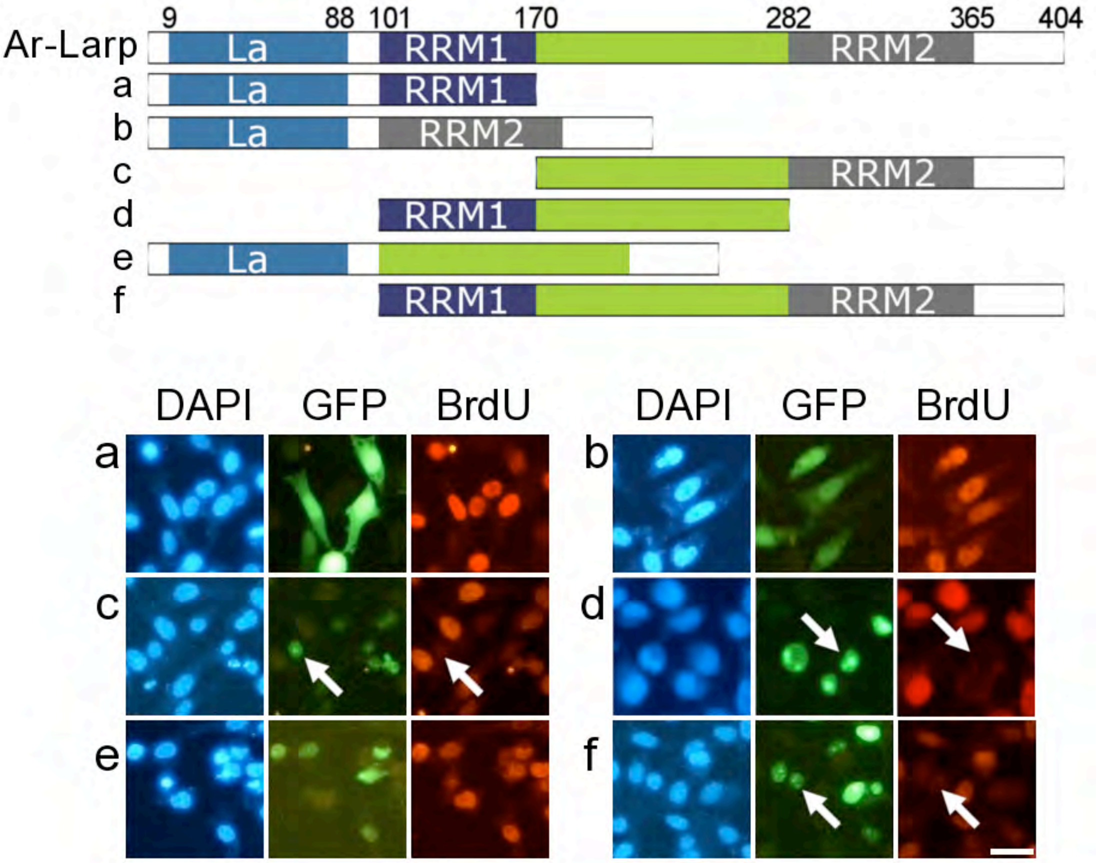

Fig. S7 Analysis of mitosis of HeLa cells overexpressing Ar-Larp mutants by BrdU-incorporation assay. Six mutants (a-f) were constructed as shown in the schematic representation. Blue, nuclei counterstained with DAPI. Green, GFP-fused Ar-Larp mutants. Red, incorporated BrdU detected with rhodamine. Scale bar = 100µm.

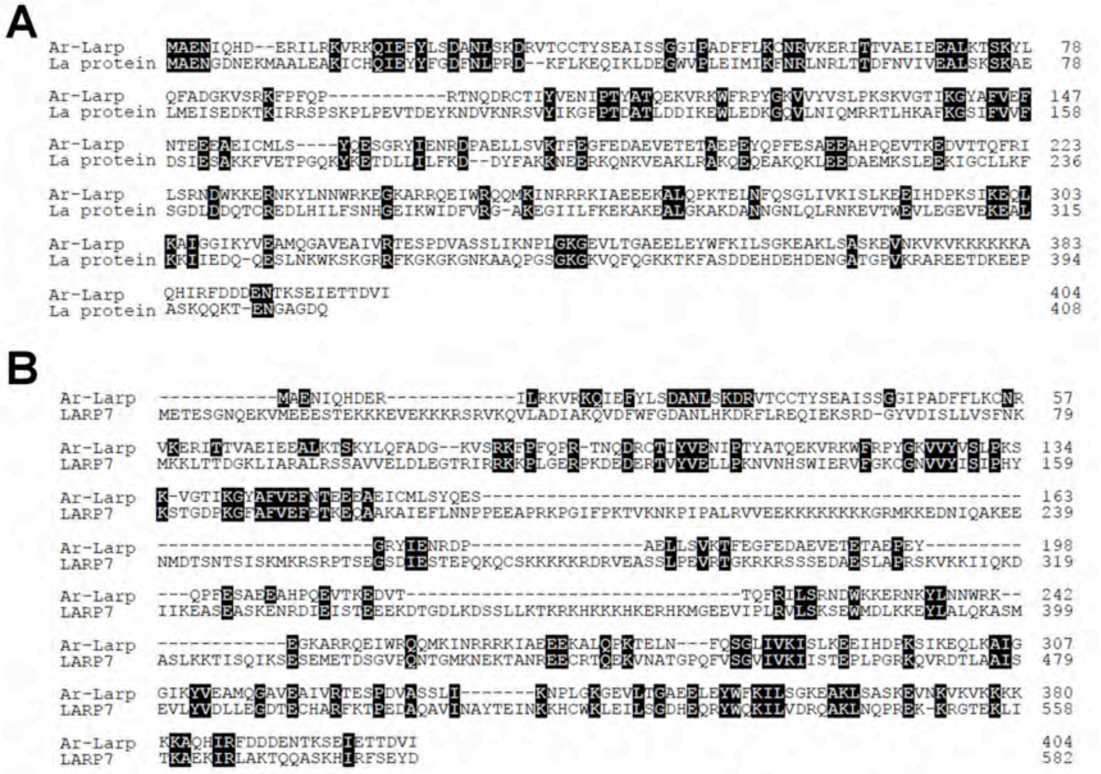

Fig. S8 Sequence alignment of Ar-Larp with human La protein (A) and LARP7 protein (B). Identical amino acid residues are shown with black background. The amino acid numbers were indicated. Ar-Larp shares only 18% and 17% sequence identities to human La and Larp7, respectively.

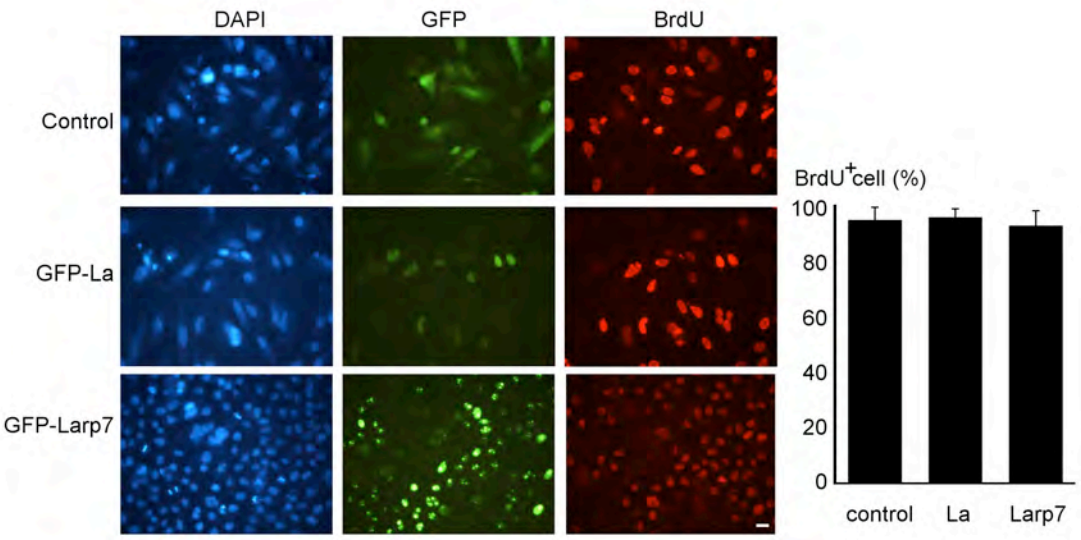

Fig. S9 Analysis of mitosis of HeLa cells overexpressing human La protein and LARP7 by BrdU-incorporation assay. Blue, nuclei counterstained with DAPI. Green, GFP-fused La and LARP7 proteins. Red, incorporated BrdU detected with rhodamine. The ratio of the BrdU positive cells was shown in the histogram. Scale bar = 50μm.

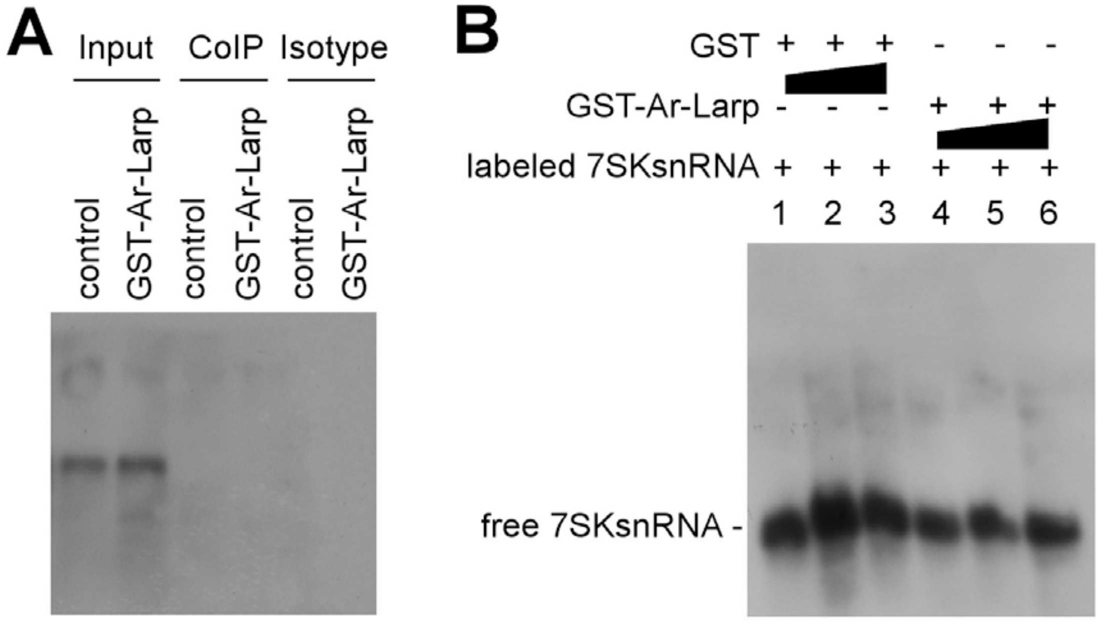

Fig. S10 The affinity of Ar-Larp to 7SKsnRNA was analyzed by CoIp and EMSA. (A) Detection of Ar-Larp-7SKsnRNA complexes by co-IP using an anti-Ar-Larp antibody and probes specific to 7SKsnRNA. Northern blot analysis of 7SKsnRNA from the pellets (co-IP) or total extract fractions (Input) of HeLa cells overexpressed GFP-Ar-Larp or GFP only (isotype antibody was used as a control). (B) EMSA analysis of the affinity between Ar-Larp and 7SKsnRNA. 7SKsnRNA (5 μg per lane) were transcript by labeled with DIG using the DIG RNA Labeling Kit. Lanes 1–3 contain increasing amounts (0.5–1 μg) of GST; Lanes 4–6 contain increasing amounts (0.5–1 μg) of GST-fused Ar-Larp. Ar-Larp-7SKsnRNA complexes were detected by Northern blotting (GST was used as control).

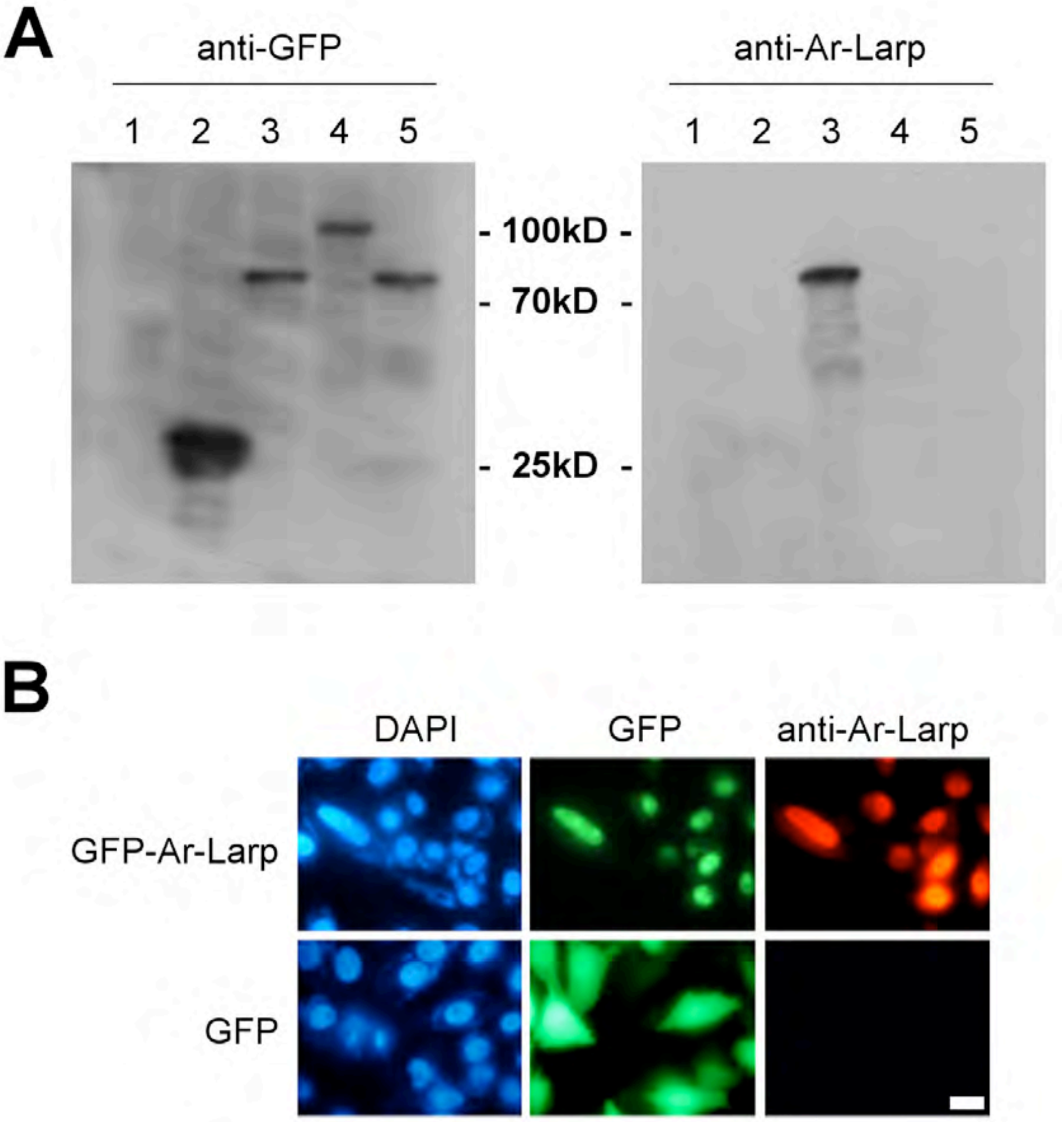

Fig. S11 Proteins in HeLa cells were detected by anti-Ar-Larp antibody. (A) Protein extracts from HeLa cells were detected by anti-GFP antibody and anti-Ar-Larp antibody. 1, HeLa cells without transfection; 2, HeLa cells overexpressing GFP; 3, HeLa cells overexpressing GFP fused human La protein; 4, HeLa cells overexpressing GFP fused Ar-Larp; 5, HeLa cells overexpressing GFP fused LARP7. (B) Immunofluorescence analysis in HeLa cells was performed using anti-Ar-Larp antibody. Blue, nuclei counterstained with DAPI. Green, GFP and GFP-fused Ar-Larp. Red, immunofluorescence signal detected with rhodamine. Scale bar = 100μm.

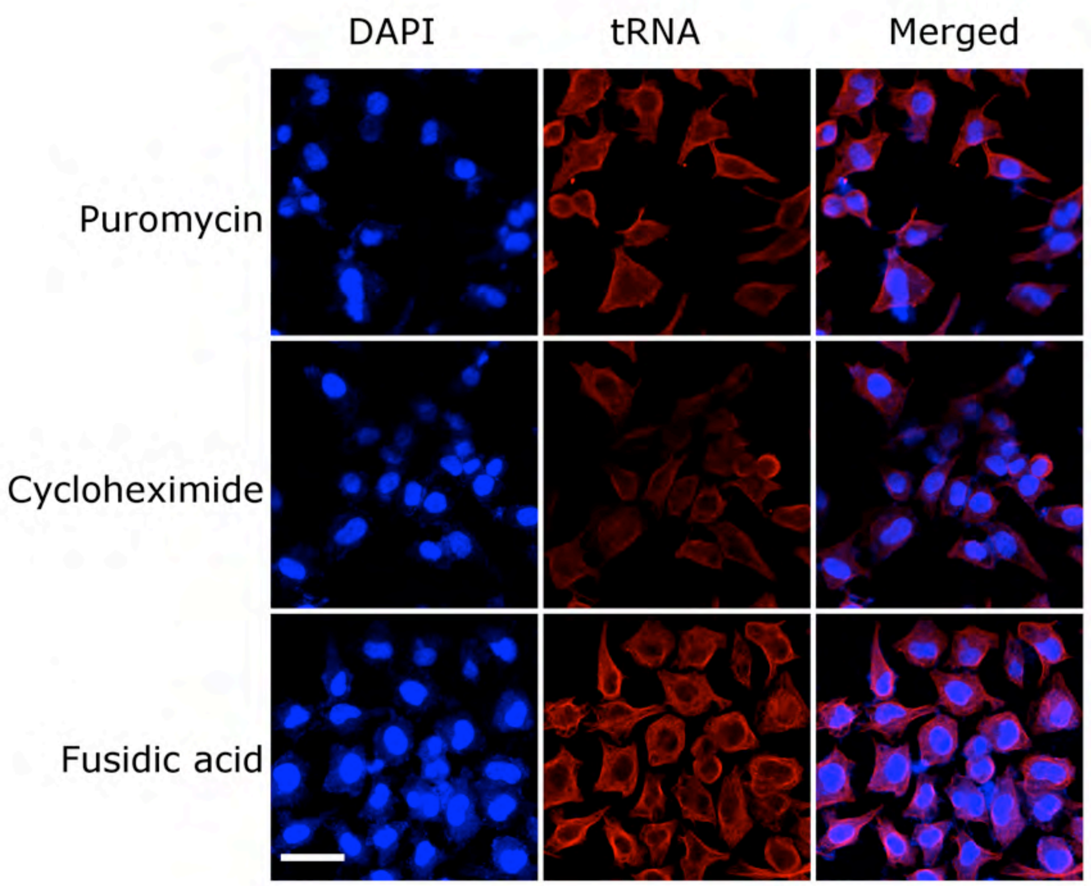

Fig. S12 The compareson of tRNA distribution of MKN45 after Ar-larp overexpressing and treated with general protein synthesis inhibitors. The tRNAs (red) were detected by FISH using oligonucleotide probes complementary to the tRNA sequences. Blue, nuclei counterstained with DAPI. Scale bar = 50μm.

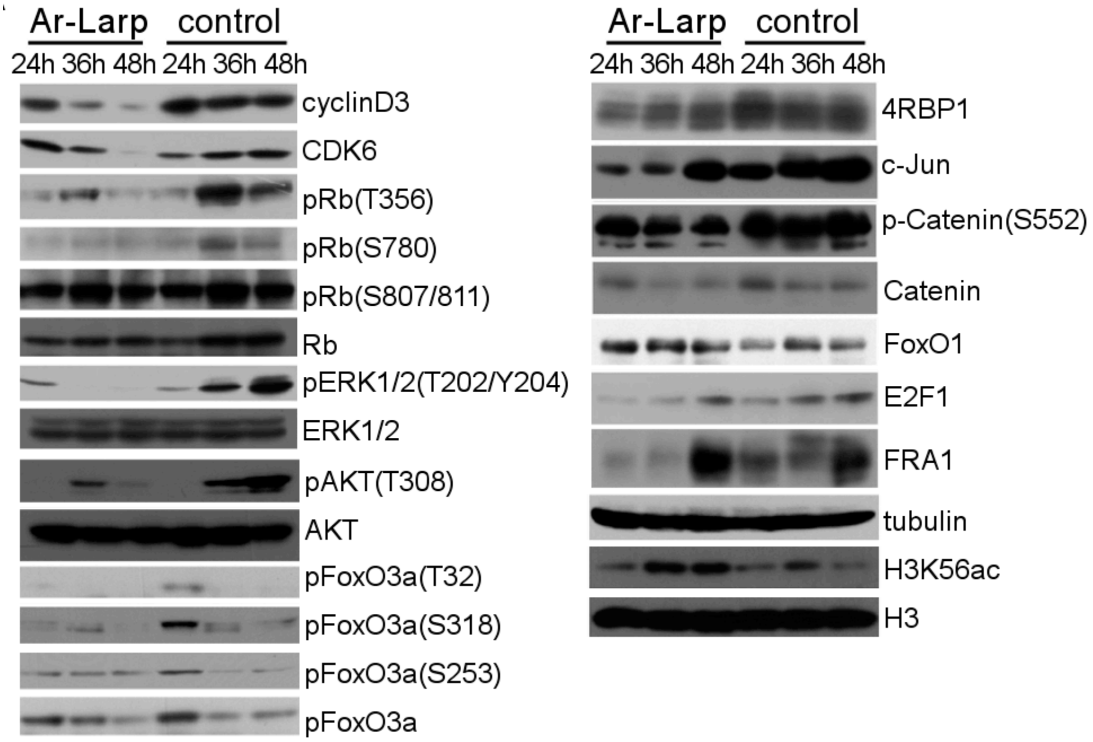

Fig. S13 Analysis of signaling pathways in Ar-Larp-induced cell cycle arrest. Western blot analysis of Cyclin D3 and CDK6 and related signaling pathways in HeLa cells 24, 36 and 48 hrs after GFP-fused Ar-Larp (GFP-Ar-Larp) or GFP only (Control) gene transfection. Histone H3 (H3) and α-tubulin/GAPDH served as loading controls.

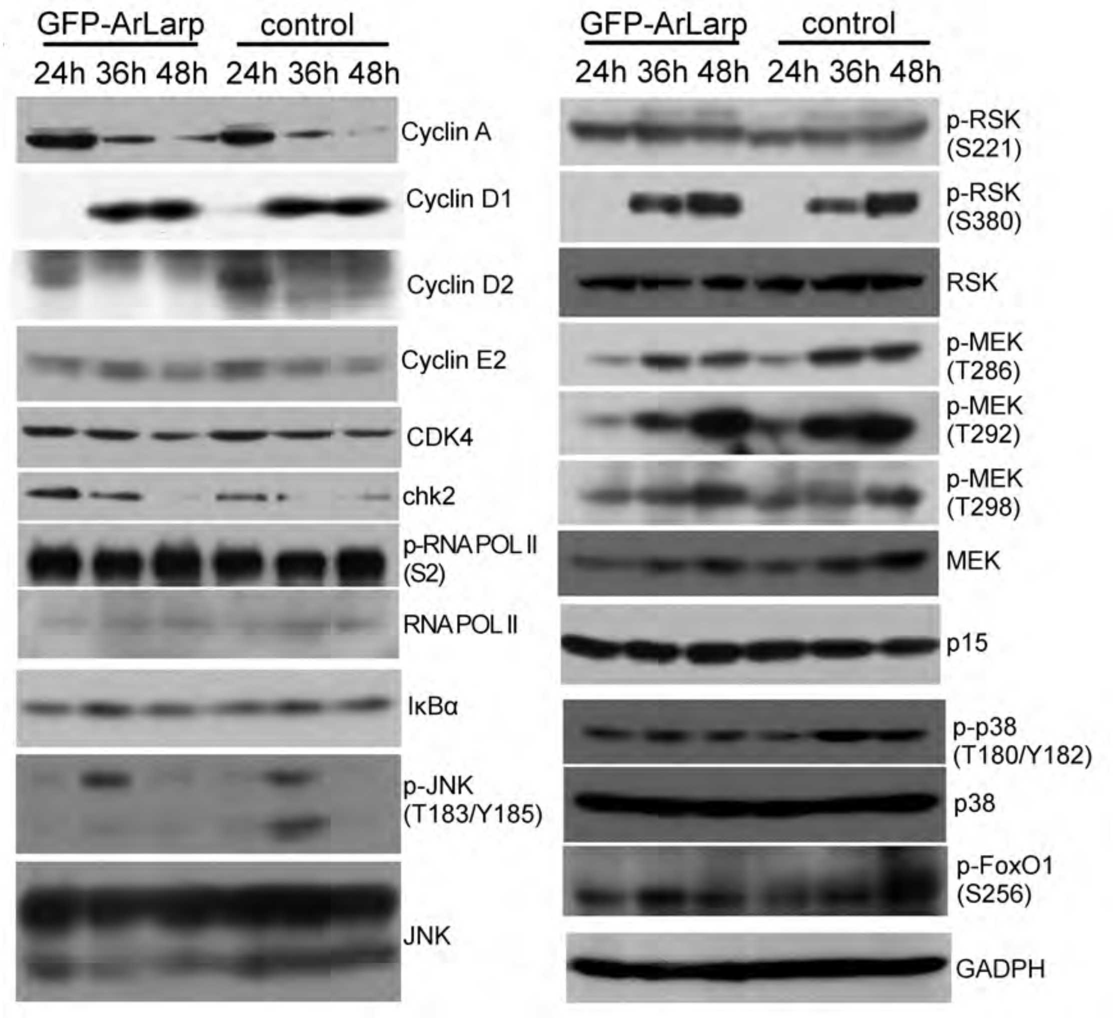

Fig. S14 Western blot analysis of cell cycle regulation related signaling pathways in HeLa cells 24, 36 and 48 hrs after GFP-fused Ar-Larp (GFP-Ar-Larp) or GFP only (Control) gene transfection. Histone H3 (H3) and α-tubulin/GAPDH served as loading controls.

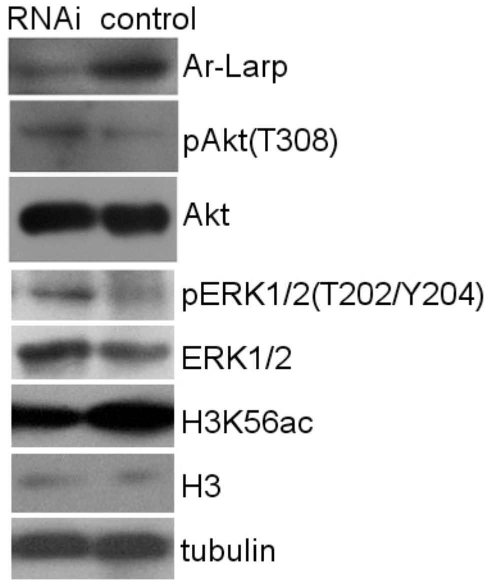

Fig. S15 Western blot analysis of pAkt (T308), pERK1/2 (T202/Y204) and H3K56ac in diapause embryos of *Artemia* after Ar-Larp RNAi treatment. RNAi: injection with 800 ng Ar-Larp-dsRNA; control: injection with 800 ng GFP-dsRNA; Histone H3 (H3) and α-tubulin/GAPDH served as loading controls.
