# Supplementary material for: An La-related protein controls cell cycle arrest by nuclear retrograde transport of tRNAs during diapause formation in Artemia
Source: BMC Biol. 2016 Mar 3;14:16. doi: 10.1186/s12915-016-0239-4 (PMC4778291; doi:10.1186/s12915-016-0239-4)
Supplement: Additional file 2: — Supplementary methods. (DOC 65 kb) [file 12915_2016_239_MOESM2_ESM.doc]

**Supplementary File 2: Supplementary Methods**

**An La-related Protein Controls Cell Cycle Arrest by Nuclear Retrograde Transport of tRNAs during Diapause Formation in *Artemia***

Dian-Fu Chen^1,2^, Cheng Lin^1^, Hong-Liang Wang^1^, Li Zhang^1^, Li Dai^1^, Sheng-Nan Jia^1^, Rong Zhou^3^, Ran Li^4^, Jin-Shu Yang^1^, Fan Yang^1^, James S Clegg^5^ , Hiromichi Nagasawa^1, 6^ and Wei-Jun Yang^1,*^

^1^ College of Life Sciences, Zhejiang University, Hangzhou 310058, People’s Republic of China

^2^ Key Laboratory of Conservation Biology for Endangered Wildlife of the Ministry of Education, Zhejiang University, Hangzhou 310058, People’s Republic of China

^3^ Key Laboratory of Protein Chemistry and Developmental Biology of the State Education Ministry of China, College of Life Sciences, Hunan Normal University, Changsha, 410018, People's Republic of China

^4^ Tianjin Key Laboratory of Animal and Plant Resistance, College of Life Sciences, Tianjin Normal University, Tianjin 300387, People's Republic of China

^5^ Section of Molecular and Cellular Biology and Bodega Marine Laboratory, University of California, Davis, Bodega Bay, California 94923, USA

^6^ Department of Biological Chemistry, The University of Tokyo, Yayoi, Bunkyo, Tokyo 113-8657, Japan

^*^ Email: w_jyang@ zju.edu.cn.

**Supplementary Methods**

**Animals and Ethics Statement**

*Artemia parthenogenetica*: Diapause-destined *Artemia* were raised in 8% (w/v) artificial seawater (Blue Starfish) at 25ºC on a 4L:20D cycle, while the directly-developing *Artemia* were reared in 4% (w/v) artificial seawater on a 16L:8D cycle. Chlorella powder (Fuqing King Dnarmsa Spirulina Co. Ltd.) was supplied as brine shrimp food. 6–8 week-old BALB/c nude mice (Institution of Animal Research, Shanghai, China) were used as xenograft recipients for cancer cells. Animal experiments were approved and performed in accordance with the institutional guidelines for animal care of animal ethics committee of Zhejiang University.

**Molecular Cloning of Ar-Larp Encoding cDNA by Screening of an *Artemia* Cysts in Diapause cDNA Library**

*Artemia* cysts in diapause were homogenized in Trizol Reagent (Invitrogen, CA, USA), and the total RNA was prepared according to the manufacturer’s instructions. Poly(A)^+^ RNA was extracted using the Oligotex mRNA Purification System (Qiagen, Germany) according to the manufacturer’s instructions. cDNA was synthesized using the ZAP-cDNA Synthesis Kit (Stratagene, CA, USA). Briefly, 5 mg of mRNA was reverse transcribed in the presence of a linker-primer and the first-strand cDNA was then nick-translated into second strand cDNA. The cDNA was ligated to an EcoRI adapter, digested with XhoI, and size fractionated on a Sepharose CL-4B Column (Amersham, NJ, USA). The cDNA was then ligated to the EcoRI/XhoI digested ZAP-II vector, and the ligation reaction was packaged using Gigapack Gold Packaging Extract Kit (Stratagene, CA, USA). The probes for plaque hybridization were prepared as described below. Plaque hybridization was analyzed using the ECL Direct Nucleic Acid Labeling and Detection System (Amersham, NJ, USA) according to the manufacturer’s instructions.

Using the cDNA library as the template, a 335-bp cDNA fragment was amplified by the primer pair Ar-LarpF1 and Ar-LarpR1 (Table S1). The primers were designed according to an RNA binding protein gene that was reported to be expressed specifically in the oocytes and released cysts of diapause-destined *Artemia* by Qiu et al. [1]. The following program was used for PCR amplification: 35 cycles of 30 s at 94°C (5 min only for the first cycle), 30 s at 50°C and 30 s at 72°C (7 min only for the last cycle). The PCR product was then subcloned into pMD18-T plasmid (TaKaRa, Japan) for nucleotide sequencing (Seq1, Table S2).

A 249-bp probe for screening the *Artemia* cysts in the diapause cDNA library was then amplified using the primer pair Ar-LarpF2 and Ar-LarpR2 (Table S1). The following program was used for PCR amplification: 35 cycles of 30 s at 94°C (5 min only for the first cycle), 30 s at 56°C and 30 s at 72°C (7 min only for the last cycle). 150 ng of the PCR product was then DIG-labeled using DIG High Prime Labeling Kit (Roche, Germany) following the manufacturer’s instructions. Positive plaques from the initial cDNA library screening were isolated in a second round of screening, and the recombinant pBluescript SK(1/2) phagemids were excised from the bacteriaphage clones according to the manufacturer’s protocol (Stratagene, CA, USA). The recombinant plasmid was subsequently sequenced.

**Quantitative Real-Time PCR**

All the samples, including ovisacs in the stages of without oocytes, early oocytes, late oocytes and early embryos of diapause-destined and directly developing *Artemia*, diapause embryos, post-diapause embryos, and nauplii hatched from post-diapause cysts and also from direct development were homogenized in Trizol reagent (Invitrogen, CA, USA) and total RNA was prepared according to the manufacturer’s instructions. First-strand cDNAs of each sample were synthesized from 2 mg of total RNA using the SuperScript/First-Strand cDNA Synthesis Kit (Invitrogen, CA, USA). After reverse transcription, all real-time PCRs were performed on the Bio-Rad MiniOpticon^TM^ Real-Time PCR System (Bio-Rad, CA, USA) using the SYBR Premix ExTaq^TM^ (TaKaRa, Japan) and 200 nM of Ar-Larp-specific primers (Ar-LarprtF and Ar-LarprtR; Table S1). Relative transcript levels are presented as fold change calculated using the comparative C_T_ method as described previously [2] with α-tubulin cDNA as the internal reference. Primers for α-tubulin were TubrtF and TubrtR in Table S1. The following program was used for PCR amplification: 35 cycles of 20 s at 94°C (5 min only for the first cycle), 20 s at 56°C, 20 sec at 72°C (7 min only for the last cycle), and 15 s read plates at 78°C. Experiments were performed in triplicate and repeated independently at least twice for each sample. The results are reported as mean values ± S.D. of three independent experiments. Comparisons between groups for statistical significance were performed with a two-tailed paired Student's t-test.

***In Situ* Hybridization**

To prepare a probe for *in situ* hybridization, the open reading frame (ORF) of Ar-Larp cDNA was amplified using a pair of specific primers. The following program was used for PCR amplification: 35 cycles of 30 s at 94°C (5 min only for the first cycle), 30 s at 56°C and 2 min at 72°C (7 min only for the last cycle). The PCR segments were then subcloned into the pSPT18 vector at EcoRI and HindIII sites. The DIG-labeled sense and antisense RNA probes corresponding to the ORF of Ar-Larp were transcribed *in vitro* using the DIG RNA Labeling Kit SP6/T7 (Roche, Germany) from the EcoRI- and HindIII-linearized templates respectively. For tissue slice preparation, diapause cysts and ovisacs in late oocyte stages were removed from the diapause-destined or directly developing *Artemia* and immediately snap-frozen in liquid nitrogen. The samples were embedded in a TissueTek OCT-Compound (Sakura Finetechnical Co. Ltd, Japan), and then cut into 8-mm thick sections using a frozen ultramicrotome. Dry sections were fixed with 4% (w/v) paraformaldehyde, digested with proteinase K, and hybridized at 42°C overnight. These slices were then blocked by 1% blocking solution (Roche, Germany) and treated with the anti-DIG-alkaline phosphatase (AP) conjugate fab fragments (1:500; Roche, Germany) and visualized with the colorimetric substrates nitro blue tetrazolium chloride/5-bromo-4-chloro-3-indolyl phosphate toluidine salt (NBT/BCIP; 1:50; Roche, Germany) according to the manufacturer’s instructions. Finally, photographs were taken using an inverted microscope equipped with a Nikon Eclipse TE2000-S camera (Nikon Instruments Inc., Japan).

**Western Blot Analysis of Ar-Larp**

Rabbit anti-Ar-Larp antibody was produced by immunization with recombinant Ar-Larp protein. The open reading frame of Ar-Larp cDNA was amplified using a pair of specific primers (Ar-LarpepF and Ar-LarpepR; Table S1). The following program was used for PCR amplification: 35 cycles of 30 s at 94°C (5 min only for the first cycle), 30 s at 56°C and 2 min at 72°C (7 min only for the last cycle). The PCR segments were then subcloned into the pET-28a(+) vector (Novagen, WI, USA) with an N-terminal 6×His tag at BamHI and XhoI sites. Recombinant Ar-Larp protein was expressed in *E. coli* BL21 (DE3) and purified by Ni-NTA Agarose (Qiagen, Germany), and the anti-Ar-Larp antibody was raised in rabbit (HuaAn, China). For nuclear and cytoplasmic fractions, the extracts were prepared using NPE Nuclear and CPE Cytoplasmic Extraction Reagents (Bioteke, China) following the manufacturer's instructions. Proteins were extracted from each tissue using protein loading buffer (0.2 mM dithiothreitol, 4% (w/v) SDS, 0.2% (w/v) bromphend blue, 20% (v/v) glycerol in 0.1 mM Tris-HCl, pH 6.8). Fifty mg of proteins of each sample were separated on 12.5% SDS-PAGE gels and transferred to PVDF membranes (Milllipore, MA, USA). The membranes were incubated with anti-Ar-Larp antibody and anti-tubulin antibody (Beyotime, China) overnight at 4°C and detected using the BM Chemiluminescence Western Blotting Kit (Roche, Germany) following the manufacturer's instructions.

**RNA-Interference**

To construct the recombinant plasmid expressing dsRNA of Ar-Larp, a 657-bp cDNA fragment in the coding region of the Ar-Larp gene was amplified by PCR with primers Ar-LarpiF and Ar-LarpiR (Table S1). The following program was used for PCR amplification: 35 cycles of 30 s at 94°C (5 min only for the first cycle), 30 s at 56°C and 45 s at 72°C (7 min only for the last cycle). The PCR products were then subcloned into pET-T7 vector at XbaI and EcoRI sites. For negative control, a 359-bp GFP cDNA fragment was subcloned into pET-T7 as described [3]. The recombinant plasmids were transformed into *E. coli* HT115, and the dsRNAs were induced and purified as described [4]. Based on the results for the basic and quantitative controls, four preparations, 100, 200, 400 and 800 ng of Ar-Larp dsRNA, and 800 ng of GFP dsRNA, were injected into the body cavity of *Artemia* just before ovarian development, with 500 individuals injected for each of the five preparation types. An UltraMicroPump II equipped with the Micro4™ MicroSyringe Pump Controller (World Precision Instruments, FL, USA) was used for the microinjection. The RNAi-treated and intact *Artemia* were cultured in 8% artificial seawater under the condition of 4L:20D. The cysts produced by RNAi-treated *Artemia* were observed by light microscopy and collected for the following experiments (Real-time PCR for the RNA interference efficiency and Western blot for the pathway assay).

**Prediction of Protein Structure**

The deduced amino acid sequence of Ar-Larp was subjected to secondary and tertiary structure predictions. The 80, 70, and 84 amino acids of the La, RRM1, and RRM2 motifs, respectively, served as submitted inputs. The secondary structures were predicted using the PSIPRED server (<http://bioinf.cs.ucl.ac.uk/psipred>) with the method implemented therein [5]. The tertiary structure modeling was performed by I-TASSER (<http://zhanglab.ccmb.med.umich.edu/I-TASSER>) [6] and visualized using RasMol 2.7.5 [7].

**Recombinant Ar-Larp and Its Mutants and *In Vitro* tRNA Binding Assay**

The cDNAs of Ar-Larp and its mutants with various motif deletions were PCR-amplified using primers (Ar-LarpepF/Ar-LarpepR for Ar-Larp, Ar-LarpepF/LaeR for La motif, Ar-LarpepF/RRM1eR for La and RRM1 motifs, Ar-LarpepF/Ar-LarpepR for La and RRM2 motifs, RRM1eF/RRM1eR for RRM1 motif, RRM1eF/Ar-LarpepR for RRM1 and RRM2 motifs, and RRM2eF/Ar-LarpepR for RRM2 motif; Table S1; schematic representation of the mutants shown in Fig. S5.). The following program was used for PCR amplification: 35 cycles of 30 s at 94°C (5 min only for the first cycle), 30 s at 56°C and 2 or 1 min (2 min for Ar-Larp and 1 min for its mutants) at 72°C (7 min only for the last cycle). Each of the PCR fragments was digested and subcloned into pGEX-4T-1 vector (GE Healthcare, CT, USA) at XhoI and BamHI sites with a C-terminal GST-tag. The recombinant proteins were expressed in *E. coli* BL21 (DE3) using IPTG induction. Each recombinant protein was purified by binding to glutathione Sepharose 4B beads (Amersham, NJ, USA) according to the manufacturer’s instructions and used for the electrophoretic mobility shift assay (EMSA). For the binding assay, yeast tRNAs were incubated with recombinant GST-fused proteins at various concentrations from 0 to 10 µg in 20 μl of binding buffer (10 mM Tris pH 7.5, 50 mM NaCl, 1 mM DTT, 1 mM EDTA and 5% glycerol). Reactions were incubated at room temperature for 30 min. The reaction mixtures were then resolved on 8% nondenaturing polyacrylamide gels following electrophoresis at 100 V for 2 hrs, using 1×TAE as running buffer. The protein-bound tRNAs and the free tRNAs were analyzed by GoldView staining or specific probes (yR-ACG, yL-CAA, yK-UUU, yS-AGA and yY-GUA according to Genomic tRNA Database [<http://gtrnadb.ucsc.edu/Sacc_cere/>], Table S3) against yeast tRNAs (ytRNA^Arg^_ACG_, ytRNA^Leu^_CAA_, ytRNA^Lys^_UUU_, and ytRNA^Tyr^_GUA_) using Northern blot analysis.

For Northern blotting, RNAs were transferred to a nylon membrane (Millipore immobilon-Nyt, MA, USA) and UV cross-linked by an Uvltec crosslinker (CL-508, UK). Hybridization was performed overnight at 39°C using the DIG Easy Hyb system (Roche, Germany) following the manufacturer’s instructions. The blots were then washed at room temperature twice at low stringency (2×SSC, 0.1% SDS) for 5 min and then at 50°C twice at high stringency (0.1×SSC, 0.1% SDS) for 15 min. After washing, hybridized probes were detected by alkaline phosphatase-conjugated anti-DIG antibody and the CSPD chemiluminescent detection system (Roche, Germany).

**Coimmunoprecipitation (Co-IP)**

Both *Artemia* cysts in diapause and post-diapause were treated with 50% sodium hypochlorite for decapsulation, and stirred in FA lysis buffer (50 mM HEPES pH 7.5, 140 mM NaCl, 1 mM EDTA, 1% Triton X-100, 0.1% sodium deoxycholate, protease inhibitors and 40 U/ml RNasin) on ice for 15 min. Cell lysates were clarified by centrifugation and pre-cleared by incubation for 1-2 hrs with Protein A Sepharose Beads (Invitrogen, CA, USA). All pre-cleared supernatants were then incubated overnight at 4°C with anti-Ar-Larp antibody. The protein-tRNA complexes were immunoprecipitated using Protein A Sepharose Beads (Invitrogen, CA, USA).

Total RNA was isolated from the complex using Trizol Reagent (Invitrogen) and detected by Northern blotting using probes against *Artemia* tRNAs (tRNA^Gly^, tRNA^Tyr^, tRNA^Ser^ and tRNA^Leu^). To obtain the specific probes, *Drosophila melanogaster* tRNA sequences from Genomic tRNA Database (<http://gtrnadb.ucsc.edu/Dmela/>) were used to design primers for *Artemia* tRNAs cloning (GlyF/GlyR for RNA^Gly^, TyrF/TyrR for tRNA^Tyr^, SerF/SerR for tRNA^Ser^ and LeuF/LeuR for tRNA^Leu^, Table S1). The PCR products cloned from *Artemia* genomic DNA were ligated into the pMD18-T vector (TaKaRa) and then sequenced. According to these tRNA sequences (Seq2 to Seq5, Table S2), four probes for tRNA^Gly^_GCC_, tRNA^Tyr^_GUA_, tRNA^Ser^_UGA_ and tRNA^Leu^_CAA_ were synthesized (aG-GCC, aY-GUA, aS-UGA, and aL-CAA, Table S3).

HeLa cells transiently expressing GFP-fused Ar-Larp or GFP were lysed in EBC buffer (50 mM Tris pH 8, 120 mM NaCl, 0.5% NP-40, protease inhibitors and 40 U/ml RNasin) on ice for 30 min and used for co-IP as described above. Total RNA was isolated from the complex using Trizol Reagent (Invitrogen) and detected by Northern blotting using probes against HeLa tRNAs (hL-CAA-pre, hY-GUA-pre, hS-AGA, and hK-CUU, according to Genomic tRNA Database [<http://gtrnadb.ucsc.edu/Hsapi19/>], Table S3).

Total RNA was extracted from the coimmunoprecipitates using Trizol according to the manufacturer’s instructions. Total RNA was then separated using 8% denaturing urea polyacrylamide gel electrophoresis at 100 V for 2 h using 1×TAE as running buffer and proceed with the Northern blotting procedure as described above.

**Construction of Recombinant Plasmids for Transfection and Cell Culture**

To construct the recombinant plasmids expressing GFP-fused Ar-Larp and its motif deletion mutants and GFP-fused La and GFP-fused LARP7, the cDNAs were amplified by PCR with primers (Ar-LarpegF and Ar-LarpegR for Ar-Larp, Ar-LarpegF/LagR for La motif, Ar-LarpegF/RRM1gR for La and RRM1 motifs, Ar-LarpegF/Ar-LarpegR for La and RRM2 motifs, RRM1gF/RRM1gR for RRM1 motif, RRM1gF/Ar-LarpegR for RRM1 and RRM2 motifs, and RRM2gF/Ar-LarpegR for RRM2 motif; LaF/LaR for La protein, LARP7F/LARP7R for LARP7, Table S1; schematic representation of the Ar-Larp mutants shown in Fig. S6.). The following program was used for PCR amplification: 35 cycles of 30 s at 94°C (5 min only for the first cycle), 30 s at 56°C and 2 or 1 min (2 min for Ar-Larp and 1 min for mutants) at 72°C (7 min only for the last cycle). Each of the PCR segments was subcloned into pEGFP-C1 vector (BD Biosciences, NJ, USA) at XhoI and BamHI sites. The recombinant plasmids were then purified by Qiagen Plasmid Midi Kit (Qiagen, Germany) following the manufacturer's instructions and used for the follow transfection.

HeLa and MKN45 cells (ATCC) were maintained in DMEM medium or EMEM medium (Genom, China), respectively, supplemented with 10% FBS (Invitrogen, CA, USA) in 5% CO_2_ at 37°C. Cells were plated on coverslips, or in 6-well plates, 24 hrs before the transfection with Lipofectamine 2000 (Invitrogen, CA, USA) as recommended by the manufacturer. Typically, 4 μg of plasmid DNA was used for transfection of cells plated in 6-well plates. Analyses were performed 12–48 hrs after transient transfection with the plasmids.

**Analysis of Cell Cycle Phase and Cell Division**

HeLa cells with GFP and GFP-fused Ar-Larp gene transfection were harvested from 6-wells plates by trypsinization. *Artemia* cells were lysed using FA lysis buffer and then crushed between slides. After washing in PBS and fixing with ice-cold 70% ethanol, cells were incubated with RNaseA (100 μg/ml, Sangon, China) for 30 min at 37°C and stained 30 min at 4°C with PI (propidium iodide, 50 mg/ml, Sangon, China) before flow cytometric analysis. Analysis of DNA content and cell cycle phase was performed using the BD FACSCalibur fluorescence-activated cell analyzer (BD Biosciences, NJ, USA). The results were analyzed using Cell Quest^TM^ software (BD Biosciences, NJ, USA). Experiments were performed in triplicate and repeated independently at least twice for each sample. The results were reported as mean values ± S.D. of three independent experiments. Comparisons between groups for statistical significance were performed with a two-tailed paired Student's t-test.

Analysis of cell division was performed by BrdU assay. HeLa and/or MKN45 cells were plated on coverslips in 6-well plates and transfected by GFP or GFP-Ar-Larp or GFP-La or GFP-LARP7 for 24 hrs. They were then incubated with complete medium supplemented with BrdU (12 mM) for another 24 hrs. After fixation with 4% paraformaldehyde/PBS for 50 min at room temperature, the cells were incubated in 2 N HCl for 30 min at 37°C and treated with blocking solution (PBS with 5% normal goat serum) for 15 min. Cells were incubated with polyclonal anti-BrdU (1:1000, Sigma, MO, USA) overnight at 4°C and incubated with secondary rhodamine-conjugated mouse monoclonal antibody (HuaAn, China) for 3–4 hrs using standard conditions. Fluorescence was viewed using a widefield fluorescence microscope. The percentages of BrdU positive cells were identified counted in the representative fields. Experiments were performed in triplicate and repeated independently at least twice for each sample. The results were reported as mean ± S.D. of three independent experiments. Comparisons between groups for statistical significance were performed with a two-tailed paired Student's t-test.

Cell proliferation assay was analyzed using HeLa cells transiently expressing GFP-fused Ar-Larp or GFP alone. The cells were separated to low confluency and the division of the cells was viewed using a widefield fluorescence microscope at 24, 48, 72 and 96 hrs after transfection.

**FISH**

*Homo sapiens* tRNAs from Genomic tRNA Database (<http://gtrnadb.ucsc.edu/Hsapi19/>) were used to design probes complementary to tRNA^Leu^_CAA_, tRNA^Tyr^_GUA_, tRNA^Gly^_GCC_, tRNA^Phe^_GAA_, tRNA^Ser^_AGA_, and tRNA^Lys^_CUU_ (Table S3). All probes were labeled at their 3' end using terminal transferase and digoxigenin-UTP (Roche, Germany) following the manufacturer's instructions.

HeLa cells with GFP and GFP-fused Ar-Larp gene transfection were harvested from 6-wells plates by trypsinization. Cells were fixed in 4% paraformaldehyde/PBS for 50 min at room temperature and permeabilized in cold acetone for 3 min at -20°C followed by denaturing target tRNA at 75°C for 10 min. Cells were then incubated in prehybridization buffer (Roche, Germany) for 1 hr at 39°C. Cells were incubated overnight at 39°C in hybridization solution containing DIG-labeled oligonucleotides: hG-GCC, hF-GAA, hK-CUU, hL-CAA and hY-GUA which used to detect both mature and pre-mature tRNAs (Table S3). Cells transfected by GFP were used as negative controls. Cells were washed with SSC buffer (2×SSC at 45°C for 10 min, twice; 1×SSC room temperature for 10 min, three times; 4×SSC with 1% Triton X-100 room temperature for 10 min, and then blocked with 4×SSC containing 1% BSA) as previously described [8]. Rhodamine-conjugated anti-digoxigenin Fab fragment (Roche, Germany) was diluted according to the manufacturer's recommendation and the cells were then incubated with the diluted antibody for 2 hrs. Cell nuclei were counterstained with 0.1 µg/ml 4',6-diamidino-2-phenylindole dihydrochloride (DAPI).

**Mouse Xenograft Model**

To construct the recombinant adenovirus expressing *Ar-Larp*, the ORF of *Ar-Larp* was amplified by PCR using primers Ar-LarpadF and Ar-LarpadR (Table S1) and using plasmid pEGFP-*Ar-Larp* (this study) as a template. The recombinant adenovirus (Ad-Ar-Larp) was then constructed and purified using the Adenovirus Dual Expression Kit (TaKaRa) following the manufacturer's instructions. BALB/c nude mice were injected subcutaneously with 3×10^6^ cells in a total volume of 100 µl DMEM into the flanks, and when the tumor size reached about 100 mm^3^, 1×10^8^ plaque-forming units (pfu)/100 µl of Ad-Ar-Larp or Ad-control were injected twice a week. Tumor volumes were determined before injection by measuring in two dimensions as described previously [9]. Four weeks later, the mice were injected with BrdU (50 mg/kg, diluted in phosphate buffered saline [PBS]), and 10 hours after this, the mice were sacrificed and tumors were harvested and paraffin embedded. The BrdU incorporation was detected as described above.

**7SKsnRNA transcription and labeling**

To prepare 7SKsnRNA for EMSA, the full length of 7SKsnRNA cDNA was amplified using a pair of specific primers (NC_000006.12). The following program was used for PCR amplification: 35 cycles of 30 s at 94°C (5 min only for the first cycle), 30 s at 56°C and 2 min at 72°C (7 min only for the last cycle). The PCR segments were then subcloned into the pSPT18 vector at EcoRI and XbaI sites. The DIG-labeled 7SKsnRNA were transcribed *in vitro* using the DIG RNA Labeling Kit SP6/T7 (Roche, Germany) from the EcoRI- and XbaI-linearized templates respectively. The EMSA procedure was performed as described before.

**Western Blotting**

In the case of *Artemia*, proteins were extracted from each stage of early and late oocytes, early and late diapause embryos, early, middle and late post-diapause embryos, early, middle and late nauplius larvae. Proteins were also extracted from HeLa cells at 24, 36, and 48 hrs after GFP-fused Ar-Larp or GFP gene transfection. The protein loading buffer (187.5 mM Tris-HCl pH6.8, 6% SDS, 30% glycerol, 150 mM DTT, 0.03% bromophenol blue) was used for the extraction. Then, 50 mg of proteins of each sample were separated on 8–12.5% SDS-PAGE gels according to the putative molecular masses of each protein and transferred to PVDF membranes (Milllipore, MA, USA) for Western blotting analysis. The membranes were incubated with the antibodies overnight at 4°C and detected using the BM Chemiluminescence Western Blotting Kit (Roche, Germany) following the manufacturer's instructions. Histone H3, α-tubulin and GAPDH served as the loading controls.

**SI References**

1. Qiu Z, Tsoi SC, MacRae TH. Gene expression in diapause-destined embryos of the crustacean, *Artemia franciscana*. Mech Dev. 2008,124: 856–867.
2. Livak KJ, Schmittgen TD. Analysis of relative gene expression data using real-time quantitative PCR and the 2-ΔΔCT Method. Methods. 2001,25: 402–408.
3. Dai JQ, Zhu XJ, Liu FQ, Xiang JH, Nagasawa H, et al. Involvement of p90 ribosomal S6 kinase in termination of cell cycle arrest during development of *Artemia*-encysted embryos. J Biol Chem. 2008,283: 1705–1712.
4. Timmons L, Court DL, Fire A. Ingestion of bacterially expressed dsRNAs can produce specific and potent genetic interference in *Caenorhabditis elegans*. Gene. 2001,263: 103–112.
5. Jones DT. Protein secondary structure prediction based on position-specific scoring matrices. J Mol Biol. 1999,292: 195–202.
6. Zhang Y. I-TASSER server for protein 3D structure prediction. BMC Bioinformatics. 2008,9: 40.
7. Sayle RA, Milner-White EJ. RASMOL: biomolecular graphics for all. Trends Biochem Sci. 1995,20: 374.
8. Sarkar S, Hopper AK. tRNA nuclear export in *Saccharomyces cerevisiae*: in situ hybridization analysis. Mol Biol Cell. 1998,9: 3041–3055.
9. Iwahori K, Serada S, Fujimoto M, Nomura S, Osaki T, et al. Overexpression of SOCS3 exhibits preclinical antitumor activity against malignant pleural mesothelioma. Int J Cancer. 2011,129: 1005–1017.
10. Bousquet-Antonelli C, Deragon JM. A comprehensive analysis of the La-motif protein superfamily. RNA. 2009,15: 750–764.
