# Supplementary material for: An La-related protein controls cell cycle arrest by nuclear retrograde transport of tRNAs during diapause formation in Artemia
Source: BMC Biol. 2016 Mar 3;14:16. doi: 10.1186/s12915-016-0239-4 (PMC4778291; doi:10.1186/s12915-016-0239-4)
Supplement: Additional file 3: Table S1. — Primes used in this study. Table S2. Sequences obtained in this study. Table S3. Oligonucleotides used in this study. (DOC 74 kb) [file 12915_2016_239_MOESM3_ESM.doc]

**Supplementary File 3: Tables S1 – S3**

**An La-related Protein Controls Cell Cycle Arrest by Nuclear Retrograde Transport of tRNAs during Diapause Formation in *Artemia***

Dian-Fu Chen^1,2^, Cheng Lin^1^, Hong-Liang Wang^1^, Li Zhang^1^, Li Dai^1^, Sheng-Nan Jia^1^, Rong Zhou^3^, Ran Li^4^, Jin-Shu Yang^1^, Fan Yang^1^, James S Clegg^5^ , Hiromichi Nagasawa^1, 6^ and Wei-Jun Yang^1,*^

^1^ College of Life Sciences, Zhejiang University, Hangzhou 310058, People’s Republic of China

^2^ Key Laboratory of Conservation Biology for Endangered Wildlife of the Ministry of Education, Zhejiang University, Hangzhou 310058, People’s Republic of China

^3^ Key Laboratory of Protein Chemistry and Developmental Biology of the State Education Ministry of China, College of Life Sciences, Hunan Normal University, Changsha, 410018, People's Republic of China

^4^ Tianjin Key Laboratory of Animal and Plant Resistance, College of Life Sciences, Tianjin Normal University, Tianjin 300387, People's Republic of China

^5^ Section of Molecular and Cellular Biology and Bodega Marine Laboratory, University of California, Davis, Bodega Bay, California 94923, USA

^6^ Department of Biological Chemistry, The University of Tokyo, Yayoi, Bunkyo, Tokyo 113-8657, Japan

^*^ Email: w_jyang@ zju.edu.cn.

**Table S1.** Primes used in this study.

| Primers | Sequences (5’ to 3’) |
| --- | --- |
| Ar-LarpF1 | ATGTCTCCCTTCCAAAATCAAA |
| Ar-LarpR1 | ATCCTAAACTGAGTGGCGACAT |
| Ar-LarpF2 | AGCAGGGACAATTAAAGGATAT |
| Ar-LarpR2 | GCGACATCCTCTTTTGTGACTT |
| Ar-LarprtF | CCATCTCAAGTGGAGGTATT |
| Ar-LarprtR | CATCTATCCTGGTTTGTTCG |
| tubrtF | TCTACTGCCGTTGTTGAGCC |
| tubrtR | ATGGAGGAAACGATTTGACC |
| Ar-LarpepF | ***CGCGGATCC***ATGGCAGAAAATATCCAACAT |
| Ar-LarpepR | ***CCGCTCGAG***TTATCAAATCACGTCTGTTGTTTC |
| Ar-LarpiF | ***GCTCGAG****A*ACCTCGAACAAACCAGGATAGA |
| Ar-LarpiR | ***GGAATTC***TATTTTATGCCGCCAATAGCTT |
| LaeR | ***CCGCTCGAG***TTAGCATCTATCCTGGTTTGTTCG |
| RRM1eR | ***CCGCTCGAG***TTAGCTTTGGAAGTTTAGTTCCGT |
| RRM1eF | ***CGCGGATCC***TTTCCTTTTCAACCTCGAACA |
| RRM2eF | ***CGCGGATCC***GACCCAGCGGAGCTGTTATCA |
| Ar-LarpegF | ***CCGCTCGAG***AGATGCCACTTGCAAATTTGC |
| Ar-LarpegR | ***CGCGGATCC***TCACTTCACAGAGCCAAGTTCGG |
| LaF | ***CCGCTCGAG***AGATGGCTGAAAATGGTGATT |
| LaR | ***CGCGGATCC***CTACTGGTCTCCAGCACC |
| LARP7F | ***CCGCTCGAG***AGATGATCCCTAACATAGAAGG |
| LARP7R | ***CGCGGATCC***TCAATCATATTCAGAAAATCTT |
| LagR | ***CGCGGATCC***TTACTAGCATCTATCCTGGTTTGT |
| RRM1gR | ***CGCGGATCC***TTACTATCCTAATGGATTTTTGAT |
| RRM1gF | ***CGCGGATCC***TTTCCTTTTCAACCTCGAACA |
| RRM2gF | ***CGCGGATCC***GACCCAGCGGAGCTGTTATCA |
| Ar-LarpadF | ***CCCATCGAT***ATGGTGAGCAAGGGCGAGGAGC |
| Ar-LarpadR | ***CCCATCGAT***TCAAATCACGTCTGTTGTT |
| GlyF | TGCATCGGCCGGGAATCG |
| GlyR | GCATCGGTGGTTCAGTGG |
| TyrF | TCCTTCGAGCCGGATTTG |
| TyrR | CCTTCGATAGCTCAGTTG |
| SerF | CGCAGTCGGTAGGATTCG |
| SerR | GCAGTCGTGGCCGAGCGG |
| LeuF | TGGCAGCGGTGGGATTCG |
| LeuR | GGTAGCGTGGCCGAGCGG |

**Table S2.** Sequences obtained in this study.

| Name | Sequences (5’ to 3’) |
| --- | --- |
| Seq1 | GGAAAGGTAGTTTATGTCTCCCTTCCAAAATCAAAAGCAGGGACAATTAAAGGATATGCTTTTGTTGAGTTCAATACAGAGGAAGAAGCAGAACGGTGCAAGGCGTCCCATCAAGAGTCTGGGATGTATATTGAAAACCGTGATCCAGCGGAGCTGCTATCAGTTAAAACATTTGAAGGATTTGAAGATCATGAGGGTGAAACTGAAACAGCGGAACCTTGAAAATCAGCCCTGTGAGAGCGCAGAAGAGGTCCATTCCGAGGAAGTCACAAAAGAGGATGTCGCCACTCAGTTTAGGATACTTAGTAGGAATGATTGGAAAAAGGAGAGAAACAA |
| Seq2 (atRNA^Tyr^_GUA_) | TCCTTCGATAGCTCAGTTGGTAAAGCGGTGGACTGTAGTGGTTAAATTTACATCCATAGGTCGCTGGTTCAAATCCGGCTCGAAGGA |
| Seq3 (atRNA^Gly^_GCC_) | CCGGGAATCGAACCCGGGTCTGGTCCGCTTCTGCTACATGTTGGCAGGGACCTATTCTACCACTAGACGATCACGGCA |
| Seq4 (atRNA^Ser^_UGA_) | TGCAGTCGTGGCCGAGCGGTTAAGGCGTTGGACTTGAAATCCAATGAGGTCTCCCCGCGTAGGTTCGAATCCTACCGACTGCG |
| Seq5 (atRNA^Leu^_CAA_) | TGTCAGGATGGCCGAGCGGTTTAAGGTGCTGGTGTTAGCCACTCTTCAATTCGGAGGCATGGGTTCGAATCCCACTTCTGACA |

**Table S3.** Oligonucleotides used in this study.

| Oligonucleotides (Short in article) | Sequences (5’ to 3’) |
| --- | --- |
| atRNA^Gly^_GCC_ (aG-GCC) | GGAATCGAACCCGGGTCTGGTC |
| atRNA^Tyr^_GUA_ (aY-GUA) | cgagccggatttgaaccagcgaccta |
| atRNA^Ser^_UGA_ (aS-UGA) | GGTAGGATTCGAACCTACGCGGGGA |
| atRNA^Leu^_CAA_ (aL-CAA) | agtgggattcgaacccatgcctccga |
| ytRNA^Arg^_ACG_ (yR-ACG) | ATTAGAAGTCAGACGCGTTGCCATTACGCCACGCG |
| ytRNA^Leu^_CAA_ (yL-CAA) | CTAAGAGATTCGAACTCTTGCATCTTACGATACCT |
| ytRNA^Lys^_UUU_ (yK-UUU) | AAAGCCGAACGCTCTACCAACTGAGCTAACAAGGA |
| ytRNA^Tyr^_GUA_ (yY-GUA) | CAGTCTTGCGCCTTAAACCAACTTGGCTACCGAGA |
| ytRNA^Ser^_AGA_ (yS-AGA) | TCGAGTCTCTCGCCTTAACCACTCGGCCATAGTGC |
| htRNA^Leu^_CAA_ (hL-CAA) | TTGAGTCTGGCGCCTTAGACCACTCGGCCATCCTGAC |
| htRNA^Tyr^_GUA_ (hY-GUA) | ctacagtcctccgctctaccgctgagctatcgaagg |
| htRNA^Gly^_GCC_ (hG-GCC) | TGCATTGGCCGGGAATCGAACCCGGG |
| htRNA^Ser^_AGA_ (hS-AGA) | GCCTTAACCACTCGGCCACGACTAC |
| htRNA^Lys^_CUU_ (hK-CUU) | TGCTCTACCGACTGAGCTAGCCGGGC |
